# Fast females, slow males: accelerated ageing and reproductive senescence in *Drosophila melanogaster* females across diverse social environments

**DOI:** 10.1093/evlett/qraf041

**Published:** 2025-11-08

**Authors:** Lauren M Harrison, Jessica Hughes, Amanda Bretman, Alexei A Maklakov, Tracey Chapman

**Affiliations:** School of Biological Sciences, University of East Anglia, Norwich Research Park, Norwich, United Kingdom; School of Biological Sciences, University of East Anglia, Norwich Research Park, Norwich, United Kingdom; School of Biology, Faculty of Biological Sciences, University of Leeds, Leeds, United Kingdom; School of Biological Sciences, University of East Anglia, Norwich Research Park, Norwich, United Kingdom; School of Biological Sciences, University of East Anglia, Norwich Research Park, Norwich, United Kingdom

**Keywords:** *Drosophila*, life history, fitness, senescence, sex differences, sexual selection

## Abstract

Females and males typically differ in lifespan, patterns of ageing, and reproduction. General explanations for variation in the magnitude of this sex-specificity remain elusive, and the role of the social environment in this context is under-explored. Sexual selection theory predicts that males should adopt a “live fast, die young” strategy, as their fitness is likely to be contingent on intense investment to achieve success in competing for potentially few matings. However, there is a growing realization that sexual selection can act on a much broader suite of “general performance” traits than only those directly related to mating competition. This, combined with frequently observed high costs of reproduction in females, makes an alternative prediction—that ageing and reproductive senescence can be high for females, and could potentially exceed what is seen in males. We tested these contrasting predictions in the fruit fly *Drosophila melanogaster*, using assays in which both sexes competed for reproductive opportunities over their lifetimes within varying socio-sexual environments. Females consistently exhibited “live fast, die young” life histories, whereas males had significantly longer lifespans and showed only limited declines in age-specific fitness and mating performance. We also identified new same-sex exposure effects—females housed with females exhibited faster reproductive ageing at a marginal lifespan cost, whereas corresponding males maintained higher courtship and activity with age, with no detectable effect on lifespan. The results highlight the crucial importance of social environments to the study of ageing and fitness. The possibility that rapid reproductive senescence is widespread in females is key to broadening our holistic understanding of the biology of sex differences in ageing and reproduction.

## Introduction

Females and males often have different lifespans, rates of ageing, and reproductive schedules (Bronikowski et al., 2022; Colchero et al., 2016; Lemaître et al., 2020b). Yet there remains a long-standing theoretical prediction that females should be the longer-lived sex because males are generally under stronger sexual selection (Bonduriansky et al., 2008; Trivers, 1972). Anisogamy underpins fundamental sex differences in life-history strategies of males and females (Schärer et al., 2012; Trivers, 1972), with males limited by the number of receptive females and the requirement to compete for mating opportunities or to fertilize eggs (Bateman, 1948). Following from this is the expectation that sex differences in lifespan, ageing, and reproductive success can result from sex differences in the allocation of limited resources that drive trade-offs between growth, reproduction, and survival (Lemaître et al., 2024; Stearns, 1989). Sexual section should therefore favor greater investment into reproduction over maintenance in males, and the opposite pattern in females (Brengdahl et al., 2018). Consistent with this idea, males typically invest more heavily into the expression of sexually selected traits than do females, often at the cost of maintaining overall body condition (Harshman & Zera, 2007; Lemaître et al., 2020a). Moreover, the pursuit of mating opportunities by males can increase mortality risks from environmental or extrinsic factors such as predation (Belwood & Morris, 1987; Godin, 2003; Tuttle & Ryan, 1981), parasitism (Cade, 1975), or intrasexual combat (Clutton-Brock & Isvaran, 2007; Lizé et al., 2014) over that of females (Bonduriansky et al., 2008). Consequently, at least outside a strongly kin-selected context, males are predicted to suffer from shorter lifespans, faster ageing, and sharper reproductive declines than are females (Harshman & Zera, 2007; Reznick, 1992).

The fundamental sex differences in gametes, mortality risks, lifespan, and reproductive costs outlined above are manifested as divergent life-history strategies, and they predict that males should generally adopt a “live fast, die young” life-history strategy in contrast to females (Reznick, 1992; Stearns, 1989). However, there are growing challenges to the generality of this view (Archer et al., 2012; Lemaître et al., 2020b; Travers et al., 2015). These stem from two main sources: an under-appreciation of the breadth of traits influenced by sexual selection, and of the importance of the social and sexual environment. For example, sex-specific selection may act on traits that are positively genetically correlated with lifespan in males, such as “general performance” traits (Lailvaux & Irschick, 2006), as well as traits directly involved in mating competition. This is expected to potentially slow senescence in males (Bonduriansky et al., 2008). The intensity and frequency of male–female mating interactions is also important in this context as it can influence the magnitude of the costs of reproduction expressed (Chapman et al., 2003) and thus sex-specific ageing rates (Maklakov & Lummaa, 2013; Promislow, 2003). For example, male sexual traits can affect female lifespan directly, through effects on females of courtship receipt and mating attempts (Flintham et al., 2018), or indirectly through effects of pheromones or other cues (Booth et al., 2022; Maures et al., 2014). Furthermore, in realistic mixed sex environments, both sexes will have the opportunity to continuously influence the costs of reproduction experienced by the other. Indeed, continuous exposure to the opposite sex is expected to elevate costs of reproduction in females above those for males, and thus shorten female lifespan. Exposure to males can also elevate female ageing by shortening the age at which age-dependent mortality rates increase (Bonduriansky, 2014). Therefore, the existence of high costs of reproduction and elevated rates of ageing in females challenges the prevailing view and actually predicts that there should be more rapid ageing in females than males (Bonduriansky et al., 2008; Graves, 2007; Maklakov & Lummaa, 2013; Promislow, 2003). At present, direct experimental tests of these contrasting predictions are remarkably limited. In particular, we lack studies that also include effects due to social and environmental variation, both of which have significant, and under-appreciated, effects on sex differences in lifespan and ageing (Austad & Fischer, 2016; Kawasaki et al., 2008).

The broader sociosexual environment can influence the costs and benefits of reproductive investment in a sex-specific manner. For males, the presence of same sex rivals can increase investment into competitive traits, such as sperm number, ejaculate volume and composition, and mating duration, to combat perceived elevated sperm competition risk (Bretman et al., 2013; Cook & Wedell, 1996; Dore et al., 2021; Gage, 1991; Simmons et al., 1993; Wedell et al., 2002). Similarly, females exposed to other females can significantly alter their investment in egg production (Bailly et al., 2023; Borg et al., 2006, 2012; Fowler et al., 2022; Pilakouta et al., 2016). However, despite the potential importance of both same and opposite sex interactions on fitness, the effects of social environments are only rarely considered in studies of ageing (though see Rostant et al., 2023; Zajitschek et al., 2009). To date, we lack experimental studies that have simultaneously measured both lifespan, ageing rates, and overall fitness in both sexes under socially mixed competitive contexts.

In this study, we used the *Drosophila melanogaster* fruit fly system, which is a well-established, powerful model for studies of ageing and fitness (Bateman, 1948; Harrison et al., 2024; Piper & Partridge, 2018; Rose & Charlesworth, 1981). In this well-studied species, females are frequently suggested to live longer than males (e.g., Austad & Fischer, 2016). However, sex differences in lifespan can be modulated by environment and social factors, including diet (Chippindale et al., 1997; Magwere et al., 2004), mating status (Hoffman et al., 2021), and sex ratio (Rostant et al., 2020), such that males can outlive females. Our aim here was to test the competing predictions for which sex would show more rapid ageing and reproductive senescence under a range of socio-sexual environments. We compared, under ecologically relevant experimental designs, the fitness and ageing rates of both sexes kept alone, or exposed to same, mixed or opposite sex individuals. We predicted the emergence of sex differences across all key life-history traits—increased exposure to the opposite sex would decrease lifespan and increase the rate of ageing for females, but not for males.

## Methods

### Experimental rationale

**Figure 1.**
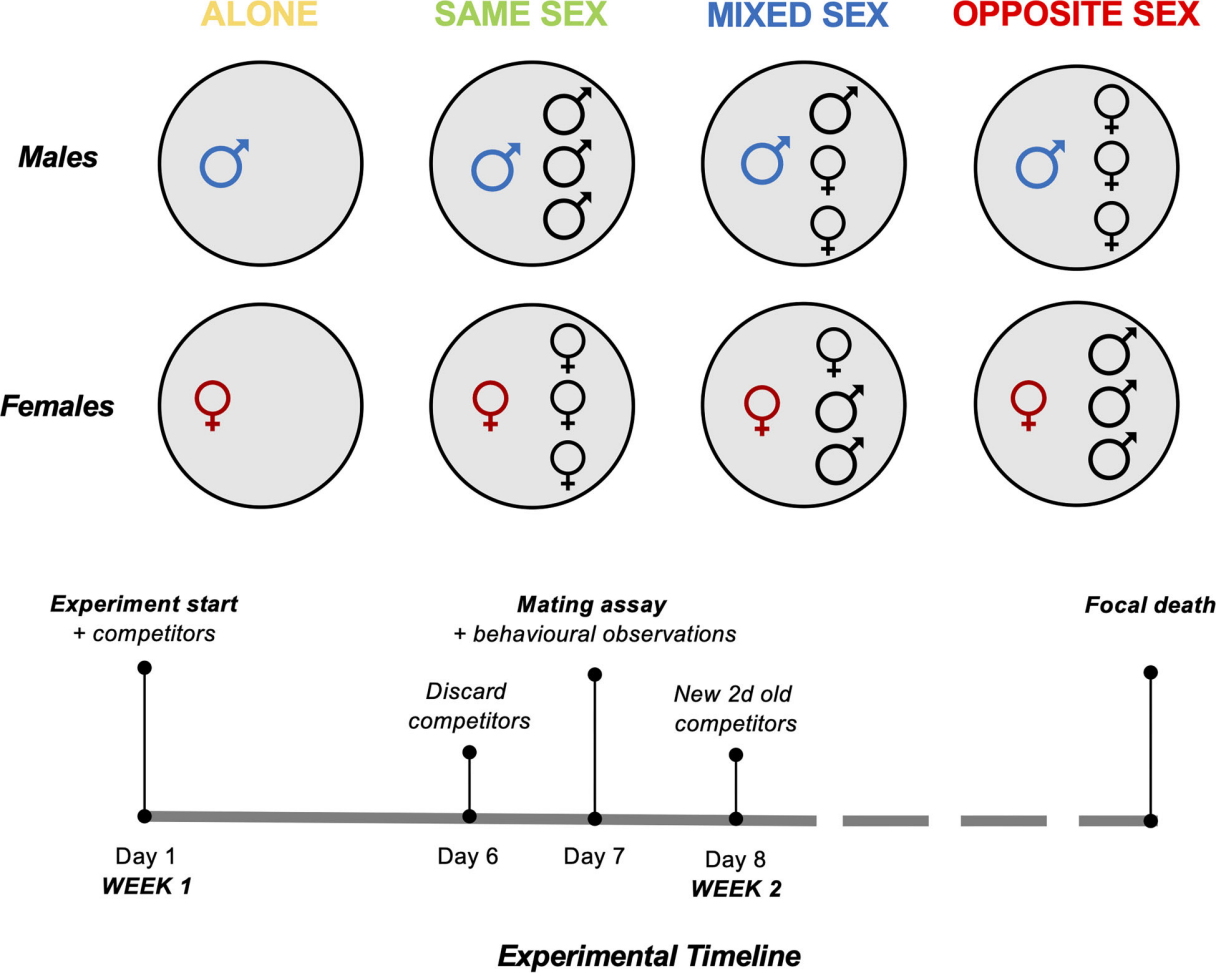
Experimental design to compare sex differences in lifespan, ageing, fitness, and behavior in response to exposure to same and opposite sex environments. Male and female *Drosophila melanogaster* were exposed to different social environments over their whole lifetimes in a 7-day repeating cycle: Alone (one focal per tube), Same Sex (one focal, three non-focals of the same sex per tube), Mixed Sex (one focal, one non-focal of the same sex, and two non-focals of the opposite sex), or Opposite Sex (one focal, three non-focals of the opposite sex). At the start of the experiment, we randomly assigned 2 days male and female flies (*N* = 160 males; *N* = 160 females; *N* = 40 flies per sex: treatment combination) to one of the four social exposure treatments (Day 1). After 5 days of social exposure, focal flies were isolated (Day 6) and then each given 24 hr of exposure in mating vials to a virgin *scarlet-*eyed partner of the opposite sex (one focal, two non-focal per vial) (Day 7). We observed and scored precopulatory behaviors in the first 2 hr following introduction in these mating vials and then left the pairs together for 24 hr. After 24 hr, focal flies were placed back into their respective social treatments with new, young non-focal flies (1–3 days at the start of each 7-day renewal) for another 5 days. Offspring from mating vials were counted after 12 days and used as a weekly indicator of individual fitness.

The experimental rationale was to expose individual “focal” males and females to varying social group treatments (*N* = 40 per sex: treatment combination; Figure 1) for 5 days out of every 7 days, then place all focal flies in a new vial on their own before giving them, on the next day, the opportunity to mate with a young virgin mating partner for 24 hr (to yield a standardized measure of age-specific reproductive output that could be compared across all treatments). Focal individuals were then returned to their 5 days social treatments and exposed to new, young competitors. This was repeated weekly until all focal individuals had died. A key distinction of our study over related previous work is that we followed mated individuals throughout their life to obtain individual-level measures of key life-history traits. The standardized weekly mating opportunity in our experimental design allowed for comparable fitness measures across the social treatments to estimate how lifetime reproductive effort might differ between the sexes and across different sociosexual environments.

Social environments (Alone, Same Sex, Mixed Sex, Opposite Sex) were chosen to vary exposure to the same and the opposite sex. Therefore, some social environments also contained eggs and larvae. Males and females might also differentially alter the physical environment (e.g., microbiome) in a manner that could then impact male and female life-history traits. We considered each of these factors to be important parts of the broader social environment, and so included this variation to bring ecological relevance to our experiment.

### Dahomey source population

Focal flies for the experiments were obtained from the outbred, laboratory-adapted wild type (WT) Dahomey population of *D. melanogaster*. This Dahomey population has been maintained at large population sizes on standard sugar yeast agar (SYA) medium (100 g brewer’s yeast, 50 g sugar, 15 g agar, 30 ml Nipagin, and 3 ml propionic acid per litre of medium) with overlapping generations to preserve high levels of naturally derived genetic variation. The use of a laboratory-adapted population that has reached genetic equilibrium is advantageous as it avoids the potential for differential gene-by-sex-by-environment interactions that could occur with flies sampled directly from the wild, which could confound social environment treatment effects. We note that the husbandry regimes of the population cages are unlikely to have exerted directional, sex-specific selection on life histories (Houle & Rowe, 2003; Magwere et al., 2004; Sgrò & Partridge, 2000, 2001).

### Fly collection and maintenance

All flies used (experimental flies and competitors/mating partners) were maintained and raised using the same culturing procedures. All social treatment and mating assay competitors carried the recessive *scarlet* eye color mutation. This phenotypic marker had been backcrossed into the Dahomey WT population >4 times, and this population was maintained in cage cultures as per the standard Dahomey cages. All flies were raised and maintained on SYA medium. Flies were kept at 25 °C in ~50%–60% relative humidity and under 12h light:12h dark conditions. Eggs were collected from yeasted grape juice agar plates divided equally into four bottles with SYA for two generations, to standardize the rearing environment and thus minimize parental effects on offspring phenotypes. F2 flies (focal individuals used in this study) were collected into bottles containing SYA within 24 hr of emergence and left for 24 hr to freely interact/mate prior to the experimental setup. Experimental flies were reared at standardized densities to minimize any environmentally determined variation in body size that could confound social treatment effects.

### Exposure of focal individuals to variation in same and opposite sex social environments

Following eclosion, 1 day old, mated focal flies were placed into individual glass vials (24 × 75 mm, containing 7 ml of SYA medium) and randomly assigned to one of the four social treatments: social isolation (Alone), with competitors of the same sex (Same Sex), in an equal sex ratio (Mixed Sex), or with competitors of only the opposite sex (Opposite Sex). All competitors were three non-virgin *scarlet* flies 1–3 days post-emergence (Figure 1). Focal flies were kept in these social treatments for 5 out of every 7 days. On the sixth day, the *scarlet* competitors were discarded, and the focal fly was moved into a new SYA vial for 24 hr prior to the mating assay. During the 5 days social exposure periods, dead *scarlet* competitors were removed and replaced with a new *scarlet* from the same cohort (same age/sex). Deaths and censors of focal flies were recorded daily until all focal files were dead. At the end of each weekly social exposure period, old vials were kept for a further 12 days for all offspring to eclose and to allow the number of offspring produced by focal individuals to be determined by genotyping offspring based on their eye color.

### Measurements of reproductive activity and fitness

**Figure 2.**
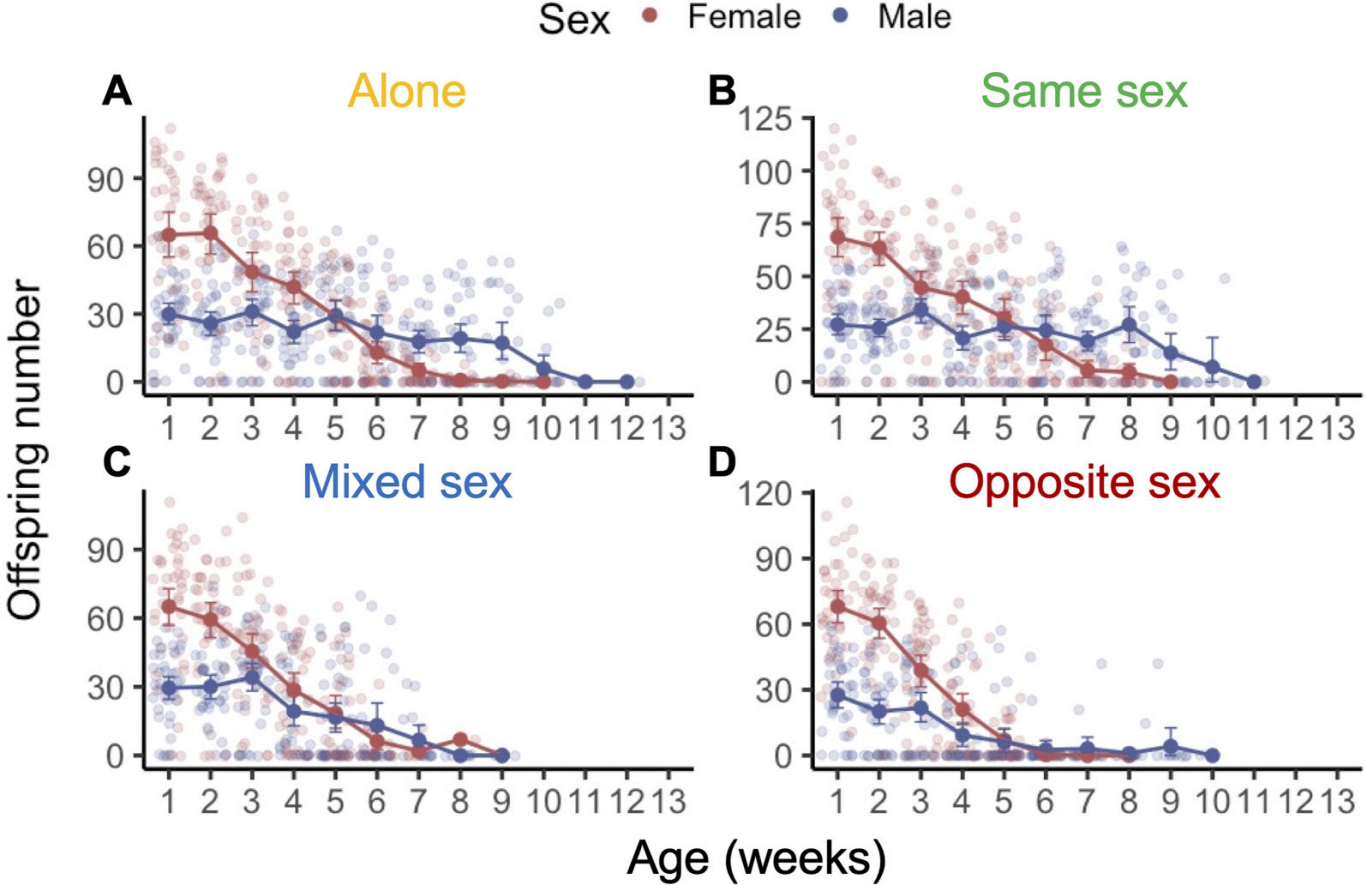
Overall reproductive senescence was greater in females in comparison to males. Comparison of female and male reproductive effort from the weekly mating exposure treatments for each of the four social exposure treatments (A–D). Plots depict the raw data, the mean, and the standard deviation for each week that we sampled reproductive output.

Every 7 days, a virgin *scarlet* mating partner (1–3 days post-emergence) was placed into each mating vial via aspiration using an electronic pooter. We then immediately observed and recorded the mating behavior of mating pairs for the first two hours of their pairing. Behavioral scans were scored anonymously with respect to social treatment. Scoring occurred at 5-min intervals using a “scanning” approach. We scored several key behaviors: (a) orientation of the male toward the female, (b) singing, (c) female chasing, (d) attempted mating, (e) mating, (f) activity that was unrelated to precopulatory behavior, and (g) inactivity. Due to logistical constraints, not all individuals were observed over the full course of their lifespan. However, all mating assays were conducted on a weekly basis even if the behavioral scoring did not take place. Mating pairs were left for 24 hr after which focal flies were moved into new social treatment SYA vials with novel, 1–3 days *scarlet* social competitors. Mating vials were kept for a further 12 days to quantify fitness from the number of emerged offspring (Figure 2).

### Post hoc measurement of additional progeny

Two of the social treatments for each sex (Mixed and Opposite Sex treatments) comprised focal males and focal females maintained with the opposite sex for 5 out of every 7 days (in addition to the 24 hr mating windows). Therefore, individuals in these treatments could have additional mating opportunities. To post hoc test whether this resulted in flies from Mixed or Opposite Sex treatments producing significantly more offspring overall, and thus whether these additional reproductive opportunities altered life-history patterns, we also counted some of the progeny samples obtained from the 5 days social treatment periods: early-life (Weeks 1 and 3), mid-life (Weeks 6 and 7), and late-life (Week 10). Due to logistic constraints, we chose to sample 5 weeks spread across the lifespan to estimate fitness during the social exposure treatments. We genotyped the adult offspring sample (*scarlet* versus WT) to assign parentage to the focal individuals and give the total number of additional offspring produced in all treatments over the 5 days social environment exposure periods. To calculate the number of extra offspring produced each day (to be comparable to the offspring counted from the 24 hr mating assay periods), we divided each of the total offspring counts by 5 (number of days in the treatment) to get a daily offspring value. For males, we then divided the daily offspring value by 2 (for males in Mixed Sex groups) or 3 (for males in Opposite Sex groups) to estimate the number of offspring produced per female per day during their social treatments. This gave us an average additional offspring count for each focal individual during their early-, mid-, and late-life periods. We chose to analyze the additional progeny in this way because the incomplete sampling across weeks, combined with some treatments having additional progeny versus no progeny, made it difficult to analyze offspring in a weekly age-specific reproduction model.

### Statistical analyses

All data analyses were conducted using R v 4.2.1 (R Development Core Team, 2020). For each of the trait and fitness models, we first fitted generalized linear mixed models (GLMMs) with different distributions and families using the *glmmTMB* (Brooks et al., 2017) package. Model fit was then assessed using the *DHARMa* (Hartig, 2020) package, and the best model was chosen from Akaike information criterion comparisons. We ran separate models for each sex. Models included treatment, week, and their interaction as fixed effects, and block was added as a random effect to account for any differences between the experimental blocks. Where there was significant zero-inflation, we ran zero-inflated models (for fitness) and hurdle models (for pre-copulatory traits). Courtship and age-specific reproduction models included quadratic age terms to account for nonlinear effects of age, and all age-related models included fly ID as a random effect to account for repeated individual measures. Pre-copulatory trait data were overdispersed, so we included an observation-level random effect in these models (Harrison, 2014). There was significant zero-inflation present in the reproduction and behavioral models for both sexes. We ran negative binomial zero-inflated and hurdle models that included the interaction between treatment and week, and the quadratic age term, in the zero-inflation model. The two models did not significantly differ, so we chose to interpret the zero-inflated model, which treats some zeros as true zeros (i.e., loss of fertility rather than absolute failure to mate). Hence, we report the probability for each treatment group to produce zero or some offspring (zero-inflated model) and the conditional model (when offspring counts >1). We ran analysis of variance (ANOVA) tests (type II for models with only fixed effects, type III for models with interaction terms) on the final models to obtain significance of fixed effects. An alpha of 0.05 was used to determine significance. Summary tables of the output for all models are provided in the Supplementary Material (Tables S1–S9).

Survival curves for each treatment and sex included censored individuals, and survival data were plotted by using Kaplan–Meier curves. We ran a Cox proportional hazards regression with sex, social treatment, and their interaction, as fixed factors in the full model. As sex was a significant predictor of mortality hazard, we ran separate models for males and females to test for effects of social treatment on mortality. Log-rank tests with a Bonferroni *p*-value adjustment were conducted to obtain pairwise comparisons for each sex-specific model. To test for sex-specific mortality and ageing patterns, we ran Bayesian trajectory analysis on the survival data separately for each sex, using the *BaSTA* (Colchero et al., 2012) package (v 1.9.5). We first used the *multibasta* function to fit several models with different mortality functions (exponential, Gompertz, Weibull, and logistic) and shapes (simple, Makeham, and bathtub). The model with the lowest deviation information criterion value was chosen as the final model. The best model for both males and females had a Gompertz mortality function and a Makeham shape parameter. Pairwise comparisons between treatments for each sex were obtained using the mean Kullback–Leibler discrepancy calibration, where values greater than 0.8 strongly indicate a true difference.

We then tested how reproductive effort might change as a function of age in both males and females across the weekly mating windows. Preliminary data visualization showed potential nonlinear age effects on reproduction, hence models included a quadratic term for age (time^2^) and its interaction with treatment. To account for greater early- than late-life reproduction, we calculated individual fitness (Λ_ind_) from the lifetable of age-specific reproduction. We constructed a Leslie matrix of the offspring counts for each week for every individual until their death. Λ_ind_ is a rate-sensitive measure of fitness that weights early reproduction more heavily than later-life reproduction so that individuals who reproduce relatively more in early life have higher values of Λ_ind_ (Lind et al., 2021). The best-fitting models had a Conway–Maxwell–Poisson distribution and included treatment as a fixed effect and block number as a random effect.

We next investigated how pre-copulatory performance in males and females might change with age and treatment using the weekly behavioral score data. As there was significant zero-inflation, we included treatment, age, and age^2^ as fixed effects (additive effects only, no interactions) in the zero-inflated component of a negative binomial hurdle model. The models were identical for males and females.

For mating effort, we counted the number of matings observed during each 2-hr behavioral assay. Mating generally occurs for 20 min; hence, multiple matings were recorded only if there was a “break” in scoring a mating between mating observations. For females, the best-fitting model had a Poisson distribution and included treatment and age, and their interaction, as fixed effects. For males, we also included treatment and age (additive effects only, no interaction) as terms in the zero-inflated component of a negative binomial hurdle model to account for significant zero-inflation.

For activity, the best-fitting model had a negative binomial distribution and included treatment and age, and their interaction, as fixed effects. For females, we also included age in the zero-inflated component of a negative binomial hurdle model to account for significant zero-inflation.

We conducted post hoc tests of whether additional progeny from the social treatments changed patterns of age-specific reproduction for males and females. We included the absolute progeny counts to the mating assay offspring counts and reran the age-specific reproduction models for males and females. The models combined counts for Weeks 1–3 (early), Weeks 4–6 (mid), and Weeks 7–12 (late).

## Results

### Overall reproductive senescence was greater in females in comparison to males

Among males that produced offspring, there was no effect of social treatment on offspring number, and no interaction between offspring number and age (ANOVA: treatment × age: *χ*^2^ = 0.26, df = 3, *p* = .97; treatment × age^2^: *χ*^2^ = 0.33, df = 3, *p* = .95; Figure 2). Thus, reproductively active males did not show evidence of reproductive senescence across any of the social environments tested. However, analyses of the data for all males showed that males from Mixed Sex and Opposite Sex treatments were significantly more likely to produce no offspring as they aged (mixed sex × age: *z* = 3.74; *p* < .001; opposite sex × age: *z* = 4.08; *p* < .001) in comparison to males held alone (alone × age: *z* = −0.13; *p* = .89) or males from the Same Sex treatment (same sex × age: *z* = 0.07; *p* = .94).

In contrast, across all social environments, females produced significantly fewer offspring as they aged, indicating the existence of widespread reproductive senescence (ANOVA: age: *χ*^2^ = 1.76, df = 1, *p* < .001; Figure 2). The likelihood of zero offspring production by Opposite Sex females (who experienced constant harassment from young males) also showed a significant increase with age (zero-inflated model: opposite sex × age: *z* = 3.76; *p* < .001). There was a strong, nonlinear effect of age on offspring production for focal females from the Same Sex groups, with these females kept with other females producing significantly more offspring in early/mid-life than females from other social treatments (conditional model: same sex × age^2^: *z* = 2.84, *p* = .005).

Reproductive effort was greatest in early life, which is the period that is key to determining an individual’s overall fitness. We calculated an individual fitness measure (Λ_ind_), which accounts for the remaining lifespan of each individual and weights early life more heavily than late life reproduction (see the *Methods* section). For males and females that reproduced at least once (Λ_ind_ > 0), we found no effect of social treatment on Λ_ind_ (ANOVA males: treatment: *χ*^2^ = 1.41, df = 3, *p* = .70; females: treatment: *χ*^2^ = 1.86, df = 3, *p* = .60; Supplementary Figures S1 and S2). This surprising result suggests that reproductive effort was greatest during early life, across all social treatments, and for both sexes. Therefore, additional reproductive effort in later life did not significantly increase an individual’s fitness.

### Pre-copulatory behavior and activity levels also declined significantly with age in females but not males

**Figure 3.**
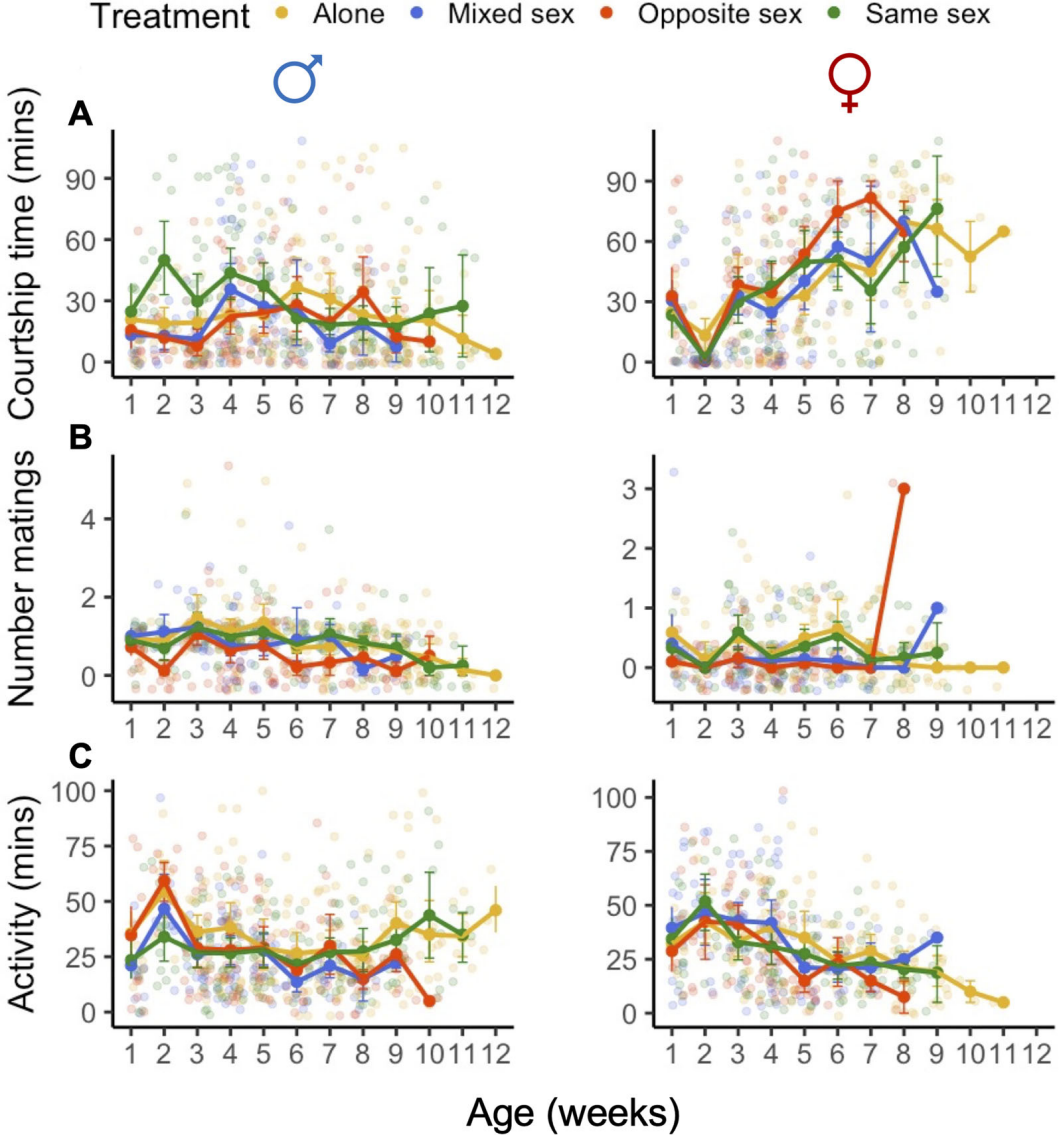
Pre-copulatory behavior and activity levels also declined significantly with age in females (right panels) but not males (left panels). (A–C) Behavioral observations made during a 2 hr observation period during the weekly mating exposure phase. Focal males and females were scored on their courtship effort (for males, male partner courtship effort for females) (A), the number of matings (B), and when focal flies were active in a manner unrelated to mating (C) (see the *Methods* section). Plots depict the raw data and the mean and standard deviation for each social exposure treatment for each week that we scored behavior during the mating assays.

We then tested whether pre-copulatory behavior also differed across the social treatments. This allowed us to examine whether males or females were adopting contrasting reproductive strategies, and whether any differences in behavior were age dependent. For courtship effort, we first combined counts for all pre-copulatory courtship behaviors (orientating, tapping/touching, singing, chasing, and attempting to mate) to obtain an index of total courtship effort during the weekly behavioral mating assays conducted across all treatments. Males from the Opposite Sex treatments were significantly more likely to refrain from courting virgin females in the mating assays in comparison to males from the other social treatments (zero-inflated hurdle model: *z* = 2.52; *p* = .01; Figure 3A). Intriguingly, in these same assays, the likelihood that males were observed courting females increased significantly with age (zero-inflated hurdle model: age: *z* = −2.73; *p* = .006; Figure 3A). Analyzing only males observed to court females, we observed that the Same Sex treatment males showed lower courtship effort with age than did males from other social treatments (conditional model: same sex × age: *z* = −1.97; *p* = .05; Figure 3A). In contrast, females showed no social experience- or age-related decline in attractiveness, and were courted by virgin males at similar levels in late- and early-life (ANOVA age: *χ*^2^ = 1.93, df = 1, *p* = .16; treatment: *χ*^2^ = 3.81, df = 3, *p* = .28; Figure 3A).

Males from all social treatments maintained their mating effort as they aged (Figure 3B). The probability of not mating in the weekly mating assays was significantly higher for Opposite Sex treatment males (zero-inflated hurdle model: *z* = 6.09; *p* < .001), and this increased significantly over time (zero-inflated hurdle model: *z* = 7.74; *p* < .001). However, among males from all social treatments that did mate, there was no significant decline in mating effort across time (ANOVA age × treatment: *χ*^2^ = 3.66, df = 3, *p* = .31; Figure 3B). Females were reluctant to mate in the weekly mating assays across all social treatments, and this did not change with age (except for a single Opposite Sex group female that mated three times in her final mating assay; ANOVA age × treatment: *χ*^2^ = 8.57, df = 3, *p* = .04; Figure 3B).

Finally, we tested whether there were age, sex, or social treatment effects on the general activity rates of flies, as a proxy for sexual and non-sexual performance/health. We first calculated a measure of activity rate by counting the number of activity scores and multiplying the total by 5 (for the 5 min period of each behavioral scan in the weekly mating assays). Only males that were kept with females (Opposite Sex treatments) showed significant age-related declines in activity rate (Figure 3C). Surprisingly, males kept with same sex rivals even slightly increased their activity levels as they aged, indicating potential positive effects of young rivals on a male’s performance (Figure 3C). Conversely, females were significantly more likely to become inactive as they aged (zero-inflated hurdle model: age: *z* = 2.05; *p* = .04; Figure 3C). Females that were active also showed the greatest activity level declines in mid-life (conditional model: age^2^: *z* = -2.93; *p* = .004).

### Males consistently outlived females and females aged rapidly when their social group contained males

**Figure 4.**
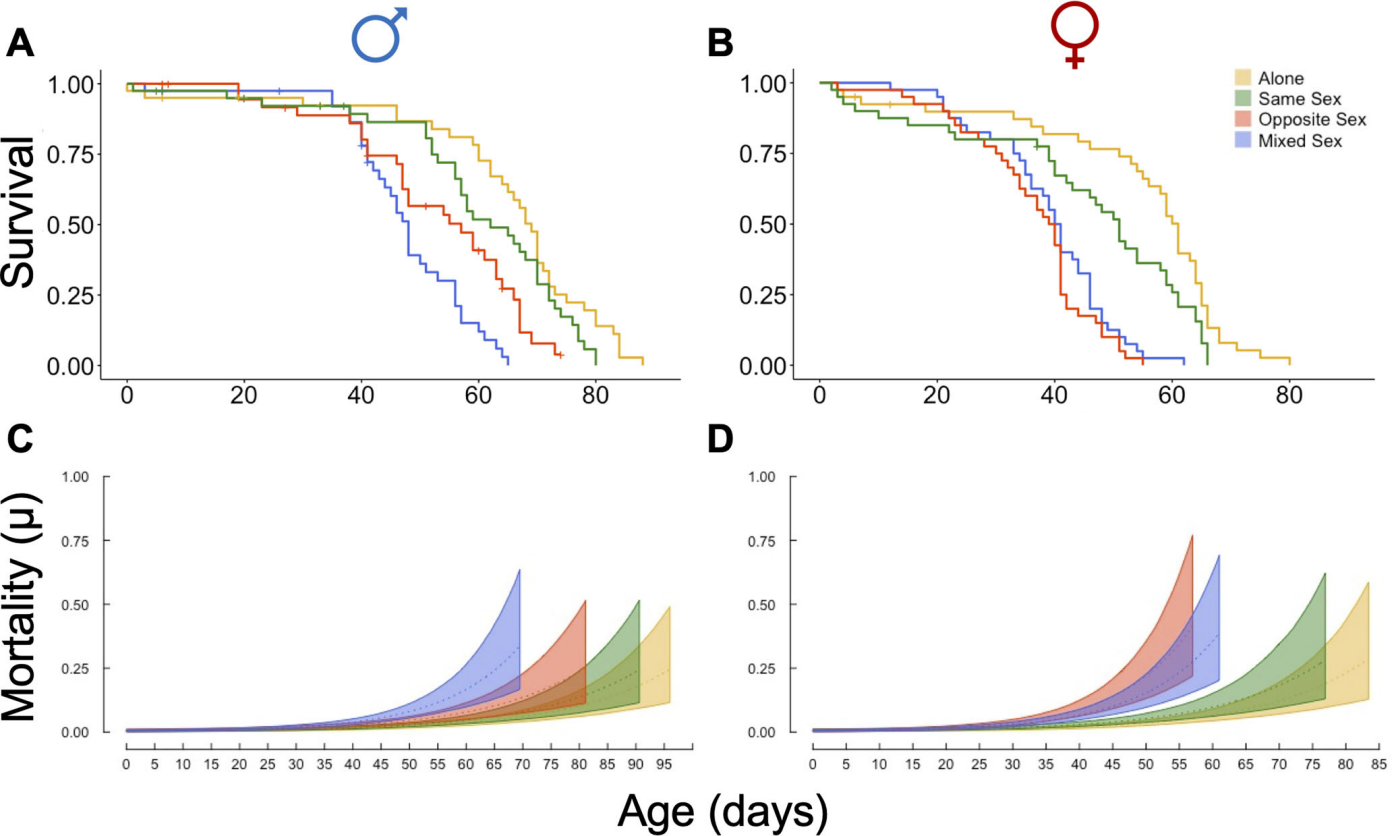
Males consistently outlived females and females aged rapidly when their social group contained males. (A, B) Survival probability for males (A) and females (B) in the different social exposure treatments. Censored individuals are indicated by a cross. (C, D) Mortality rate plots show the mean estimated mortality rate (dotted line) and 95% confidence interval (ribbon) for males (C) and females (D) for each social exposure treatment. Obtained from *BASTa* models (see the *Methods*section).

Males outlived females across all social treatments. However, exactly how social group composition influenced lifespan was sex-specific (Figure 4A). As expected, both sexes had the longest lifespans when kept mostly on their own. Exposure to same sex rivals resulted in no detectable reduction in male lifespan (log-rank test: *p* = .40; Figure 4A), whereas exposure to females significantly shortened it (Opposite Sex: log-rank test: *p* > .001). Exposure to both rivals and females had the most marked effect and significantly reduced mean male lifespan further, to just 44 days (Mixed Sex: log-rank test: *p* > .01; Table S1). For females, the presence of any males (either in Mixed or Opposite Sex groups) significantly shortened lifespan (Mixed Sex: *p* < .001; Opposite Sex: *p* < .001) in comparison to females held mostly on their own. In contrast to the findings for males, exposure to same sex competitors also significantly reduced female lifespan (*p* = .05; Figure 4A).

To test whether there were effects of the social environments on underlying ageing patterns, we compared the mortality rates of males and females. There were no significant differences in the baseline mortality rates in the different social environments across males or females (Figure S3). However, males kept in Mixed Sex groups experienced faster ageing than did males in the other social treatments (Figure 4B). For females, those kept in social groups with males (Opposite or Mixed Sex) aged significantly more rapidly than did females kept alone or with other females (Figure 4B). These results show that exposure to males accelerated senescence in females and increased the rate of ageing in males only in environments in which there was also competition for females.

### Sex-specific plasticity in reproductive strategies in response to same versus opposite sex exposure

**Figure 5.**
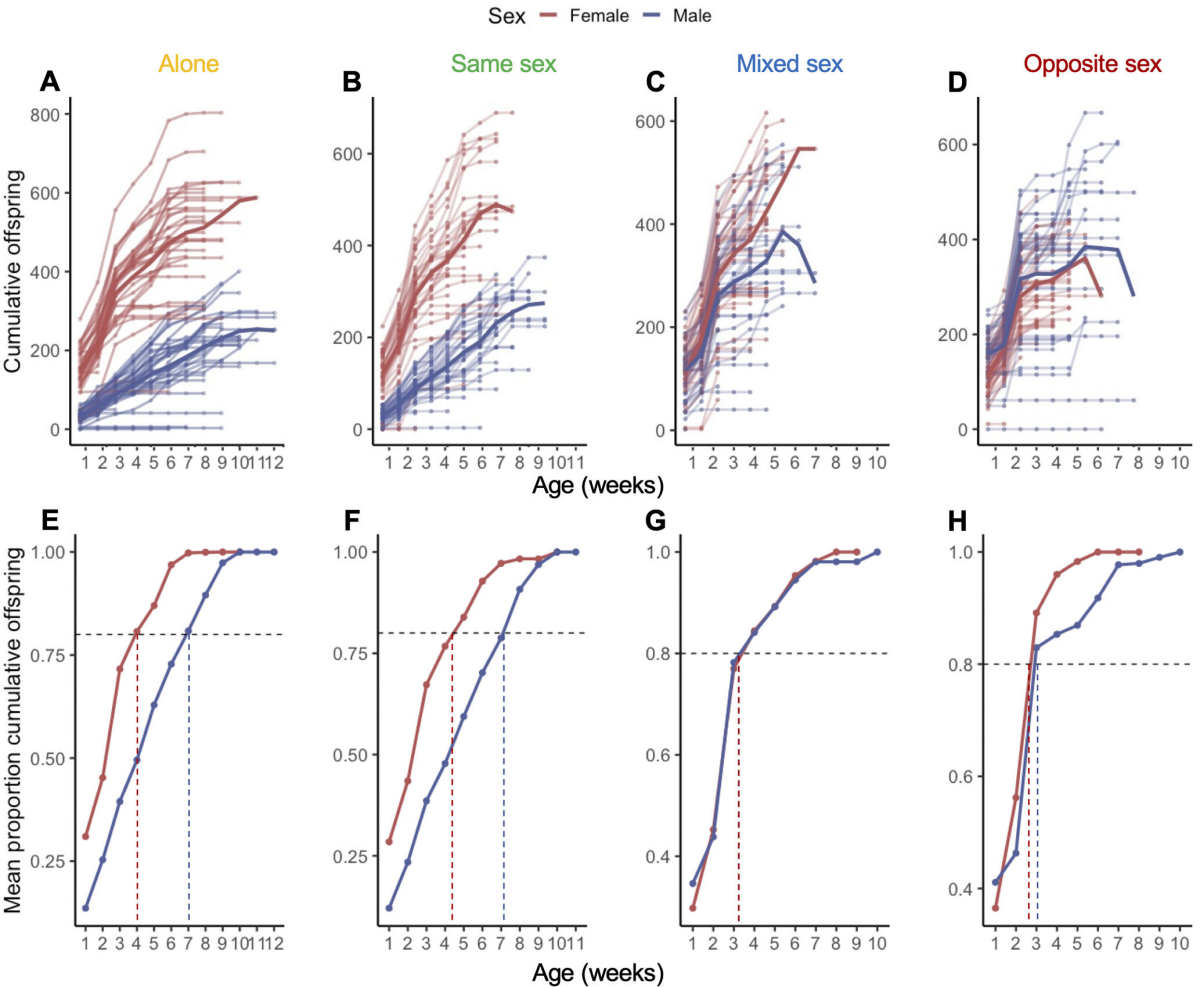
Males in all social treatments were the same age, or older, than females when they achieved almost all of their total lifetime fitness, suggesting that males can shift their reproductive effort to maximize reproductive output earlier in life in response to available mating opportunities. (A–D) Cumulative offspring for males and females produced during the weekly mating treatment phase when pooled with genotyped offspring from the social exposure phase at Weeks 1, 3, 6, 7, and 10. Individuals are plotted with the mean for males and females shown as a dark solid line for each social exposure treatment. (E–H) Comparison of the mean cumulative proportion of offspring produced by males and females. Vertical dotted lines indicate the average age at which females and males reached 80% of their total fitness (indicated by horizontal dotted line) within each of the social exposure treatments.

Males that were exposed to females in their social treatments (i.e., males in Mixed or Opposite Sex treatments) had extra mating opportunities outside of the weekly mating assays. Hence, these males had the opportunity to produce significantly more offspring than the Alone or Same Sex treatment. We tested for this by counting the offspring produced in between the weekly mating assays by focal flies at five different time points: early-life (Weeks 1 and 3), mid-life (Weeks 6 and 7), and late-life (Week 10). As expected, the Mixed Sex and Opposite Sex treatment males produced significantly more additional offspring during early-life (Weeks 1 and 3) than did males that had no additional mating opportunities (Alone or Same Sex treatment males; Figure 5A). There was also a strong, additive effect of the number of non-focal females in the social treatment on the number of additional offspring produced. Males in the Opposite Sex treatment (exposed to three young females in the social treatments) produced significantly more offspring than did males in the Mixed Sex treatment (two young females and one rival male) and with Mixed Sex males producing double the offspring of males in the Alone or Same Sex treatments (exposed to a single female for only 24 hr each week). These findings showed that males could almost double their fitness in early life with each additional mating partner. In contrast, females did not significantly benefit from additional mating opportunities outside of the weekly mating assays (Figure 5A). Females from all social treatments produced similar numbers of offspring during their social exposure periods, regardless of the number of males to which they were exposed.

We then post hoc tested whether any additional offspring produced outside of the weekly mating assays significantly altered the pattern of life histories in males or females. To do this, we calculated the cumulative proportion of total offspring produced to determine, for each treatment, the average age by which ≥80% of lifetime total offspring had been produced. This revealed that males in all social treatments were the same age, or older, than females when they achieved 80% of their total fitness (Figure 5B). Alone and Same Sex treatments were, on average, 7 weeks old when they achieved 80% of their total fitness (Figure 5B). However, Mixed and Opposite Sex treatment males achieved 80% of their total fitness much more rapidly, at around 3 weeks old, with their ~7 weeks of remaining lifespan resulting in no significant additional fitness gains (Figure 5B). Conversely, females in all social treatments consistently achieved 80% of their total fitness at 3–4 weeks of age (Figure 5B). These findings suggest that males exhibited a spectrum of plastic life-history strategies in response to their social environment—from “live fast, die young” (Mixed and Opposite Sex males) to “live slow, die old” (Alone and Same Sex males). Hence, when potential mating opportunities increased (i.e., more available females), males invested more heavily into maximizing their fitness during early life at the expense of reproduction later in life. Females, however, robustly adopted a consistent “live fast, die young” strategy that did not change with social environment nor additional mating opportunities.

## Discussion

Our results challenge the prevailing view that strong sexual selection on males should lead to more rapid ageing and reproductive senescence in males than females. By using an experimental framework that employed a range of biologically relevant sociosexual environments, we showed that females experienced more rapid reproductive and actuarial senescence by mid-life than did males. The results highlighted a steep cost of early-life reproduction, elevated ageing, and rapid reproductive senescence in females across all social environments. In contrast, males consistently outlived females and maintained pre-copulatory performance and general activity levels well into old age. Taken together, our findings demonstrate that when both sexes can compete for reproductive opportunities over the course of their lifetime, females can exhibit a “live fast, die young” strategy. Our findings also support the idea that exposure to the same versus opposite sex can have strong and opposing consequences for actuarial ageing for males versus females (Rostant et al., 2023).

A major driver of the idea that males should show more rapid reproductive ageing than females is derived from evolutionary theories of ageing, which propose that increased rates of extrinsic mortality (assumed to be higher in males) should lead to accelerated rates of intrinsic mortality, which then reduces selection on late life performance (Bonduriansky et al., 2008; Williams, 1957; but see Caswell, 2007; Moorad et al., 2019). However, sexual selection can select for male traits that are positively genetically correlated with performance and lifespan. For example, sex-limited selection on mate search activity in male *Caenorhabditis remanei* nematodes resulted in the correlated evolution of longer lifespan in males only (Chen & Maklakov, 2014). Consequently, there was evolution of increased sexual dimorphism in lifespan, with males emerging as the longer living sex (Chen & Maklakov, 2014). Our findings that male *D. melanogaster* maintain their reproductive performance well into old age are consistent with the hypothesis that sex-specific selection maintains sexual dimorphism in age-specific life-history traits. Males in our study experienced no significant declines in their courtship effort, mating success, or general activity levels. The maintenance of male performance traits might increase the amount of harm inflicted on females during mating. Thus, if male general performance is maintained and extends lifespan, then male harassment should increase the costs of reproduction for females, thereby accelerating the demise of females. It is also possible that costly male harassment might be lessened in a more spatially complex environment where females can “escape” from mating interactions with males (e.g., Yun et al., 2017).

The fitness costs of exposure to the opposite sex were far greater in females than in males. Females kept with males had significantly shorter lifespans, aged more rapidly, and suffered sharp declines in reproductive output that were evident across all social environments. This is consistent with previous research reporting that negative phenotypic correlations between early-life reproductive effort and lifespan can produce sex differences in lifespan (Lemaître et al., 2015; Rice, 1996, 1998; Travers et al., 2015). Our unexpected finding of faster female than male reproductive senescence could be partly due to our population of flies being maintained under overlapping rather than discrete generations, which could, in theory, change sex-specific selection gradients compared to previous studies (e.g., Kimber & Chippindale, 2013). Overlapping generations are probably more ecologically relevant in this model organism.

Sex differences in ageing rates could also be driven by widespread sexual conflict. Interlocus sexual conflict can contribute to the accumulation of alleles with deleterious effects in old age or select for alleles that enhance early life fitness at the cost of late life fitness (Bonduriansky et al., 2008; Maklakov & Chapman, 2019). This could then contribute to a “live fast, die young” strategy in females if negative effects of male seminal fluid proteins elevate rates of female mortality (Promislow, 2003). The optimal strategy promoted by natural selection in females might then be high rates of mating in early life because females would be unlikely to survive long enough in natural populations of flies to experience the fitness costs associated with deleterious alleles expressed in late life. In support of this hypothesis, removal of sexual selection can lead to the evolution of longer intrinsic lifespan in females (Archer et al., 2015; Maklakov et al., 2007). In our study, we provided focal individuals with young competitors and mating partners each week, potentially magnifying the sex-specific effects of interlocus sexual conflict. However, our finding that rapid reproductive senescence was widespread across social treatments for females, including individuals that experienced minimal male contact (i.e., minimal interlocus sexual conflict), suggests that our experimental design was appropriate to resolve overall comparisons of male and female life history and ageing patterns.

An unexpected and striking result was the differences between males and females in the effects of same-sex competitors on several key life-history traits. For males, there was a beneficial effect of the presence of young, same sex rivals. Such males courted virgin females at a significantly higher rate, remained more active with age, and suffered no loss of lifespan when compared with males from the other social exposure treatments. In contrast, females from the same sex treatments had significantly higher fecundity, but also had significantly shorter lifespans, than females that were kept in isolation, but did not differ from the other social exposure treatments where males were present. Sex-specific effects of same-sex rival presence on lifespan have previously been reported for *D. melanogaster* (Bretman et al., 2013; Leech et al., 2019; Rostant et al., 2023), and the general observation is of negative effects of same sex competitors of the same age on male and not female lifespan. The detrimental effect of young same-sex competitors on female but not male lifespan that we observed here could partly be due to the effects of replacing non-focal competitors every week with novel, young individuals that could potentially provide older focal flies with lifespan-extending benefits (Cho et al., 2021). A key difference in our study over previous works is that all focal flies (even those spending most of their lives in social isolation) were provided with regular mating opportunities throughout their lives. Regularly mated females exposed to same sex competitors might experience additional costs associated with competition for oviposition sites (Fowler et al., 2022), which could contribute to more rapid ageing and loss of lifespan. Female lifespans are also much more sensitive to the nutritional environment than are males, with high-nutrition diets promoting higher fecundity but shorter lifespans (e.g., Chippindale et al., 1993). It is possible that, in the social environments containing more than one mated female, oviposition and subsequent larval activity changed the nutritional environment in a way that then reduced female lifespans. However, our results suggest that females exhibited higher reproductive senescence compared to males across all the social environments, including those where females were alone and, presumably, expressed reproductive costs at a lower level. Moreover, previous work using the Dahomey population has demonstrated that egg production per se has negligible effects on female lifespan (Barnes et al., 2008; Partridge et al., 1986). Further work is now required to understand the mechanisms underlying these intriguing and sex-specific plastic social effects and the full range of their potential impacts on male and female fitness.

## Conclusion

In sum, our findings clearly show that female fruit flies are “living fast and dying young” compared to their male counterparts. Strikingly, this result applies both to reproductive ageing and to actuarial senescence, and most importantly is maintained across a range of social environments that encompass different forms of sociosexual interactions. Males showed little evidence of decline in their reproductive performance with age. These results challenge the view that sexual selection should lead to accelerated male ageing and suggest that high costs of mating and reproduction may result in faster female ageing. Future studies could usefully focus on evaluating sex-specific lifespan and ageing using different taxa in diverse sociosexual environments where both sexes are allowed to interact in a biologically relevant way.

## Supplementary Material

qraf041_Supplemental_Files

## Data Availability

All data and code used for analyses have been provided as supplementary material.

## References

[bib1] Archer C., Zajitschek F., Sakaluk S., Royle N., Hunt J. (2012). Sexual selection affects the evolution of lifespan and ageing in the decorated cricket *Gryllodes sigillatus*. Evolution, 66, 3088–3100. 10.1111/j.1558-5646.2012.01673.x 23025600

[bib2] Archer C. R., Duffy E., Hosken D. J., Mokkonen M., Okada K., Oku K., Sharma M. D., Hunt J. (2015). Sex-specific effects of natural and sexual selection on the evolution of life span and ageing in *Drosophila simulans*. Functional Ecology, 29, 562–569. 10.1111/1365-2435.12369

[bib3] Austad S., Fischer K. (2016). Sex differences in lifespan. Cell Metabolism, 23, 1022–1033. 10.1016/j.cmet.2016.05.019 27304504 PMC4932837

[bib4] Bailly T., Kohlmeier P., Etienne R., Wertheim B., Billeter J. (2023). Social modulation of oogenesis and egg laying in *Drosophila melanogaster*. Current Biology, 33, 2865–2877.e4. 10.1016/j.cub.2023.05.074. 37369209

[bib5] Barnes A., Wigby S., Boone J., Partridge L., Chapman T. (2008). Feeding, fecundity and lifespan in female *Drosophila melanogaster*. Proceedings of the Royal Society B: Biological Sciences, 275, 1675–1683. 10.1098/rspb.2008.0139 PMC2452982 18430646

[bib6] Bateman A. (1948). Intra-sexual selection in *Drosophila*. Heredity, 2, 349–368. 10.1038/hdy.1948.21 18103134

[bib7] Belwood J., Morris G. (1987). Bat predation and its influence on calling behavior in neotropical katydids. Science, 238, 64–66. 10.1126/science.238.4823.64 17835656

[bib8] Bonduriansky R. (2014). The ecology of sexual conflict: Background mortality can modulate the effects of male manipulation on female fitness. Evolution, 68, 595–604. 10.1111/evo.12272 24102073

[bib9] Bonduriansky R., Maklakov A., Zajitschek F., Brooks R. (2008). Sexual selection, sexual conflict and the evolution of ageing and life span. Functional Ecology, 22, 443–453. 10.1111/j.1365-2435.2008.01417.x

[bib10] Booth L. N., Shi C., Tantilert C., Yeo R. W., Miklas J. W., Hebestreit K., Hollenhorst C. N., Maures T. J., Buckley M. T., Murphy C. T., Brunet A. (2022). Males induce premature demise of the opposite sex by multifaceted strategies. Nature Aging, 2, 809–823. 10.1038/s43587-022-00276-y. 37118502 PMC10154206

[bib11] Borg Å., Åsmul T., Bolstad G., Viken Å., Berglund A., Rosenqvist G. (2012). Interactions among female guppies (*Poecilia reticulata*) affect growth and reproduction. Ethology, 118, 752–765. 10.1111/j.1439-0310.2012.02065.x

[bib12] Borg Å., Rosenqvist G., Amundsen T., Forsgren E. (2006). Presence of same sex individuals negatively affects egg maturation in female guppies (*Poecilia reticulata*). Behaviour, 143, 747–761.

[bib13] Brengdahl M., Kimber C., Maguire-Baxter J., Malacrinò A., Friberg U. (2018). Genetic quality affects the rate of male and female reproductive aging differently in *Drosophila melanogaster*. The American Naturalist, 192, 761–772. 10.1086/700117 30444654

[bib14] Bretman A., Westmancoat J., Gage M., Chapman T. (2013). Costs and benefits of lifetime exposure to mating rivals in male *Drosophila melanogaster*. Evolution, 67, 2413–2422. 10.1111/evo.12125 23888861

[bib15] Bronikowski A. M., Meisel R. P., Biga P. R., Walters J., Mank J. E., Larschan E., Wilkinson G. S., Valenzuela N., Conard A. M., de Magalhães J. P., Duan J., Elias A. E., Gamble T., Graze R., Gribble K. E., Kreiling J. A., Riddle N. C. (2022). Sex-specific aging in animals: Perspective and future directions. Aging Cell, 21, 1–25. 10.1111/acel.13542 PMC8844111 35072344

[bib16] Brooks M., Kristensen K., Benthem K., Magnusson A., Berg C., Nielsen A., Skaug H., Mächler M., Bolker B. (2017). glmmTMB balances speed and flexibility among packages for zero-inflated generalized linear mixed modeling. The R Journal, 9, 378–400. 10.32614/RJ-2017-066

[bib17] Cade W. (1975). Acoustically orienting parasitoids: Fly phonotaxis to cricket song. Science, 190, 1312–1313. 10.1126/science.190.4221.1312

[bib18] Caswell H. (2007). Extrinsic mortality and the evolution of senescence. Trends in Ecology & Evolution, 22, 173–174. 10.1016/j.tree.2007.01.006 17287044

[bib19] Chapman T., Arnqvist G., Bangham J., Rowe L. (2003). Sexual conflict. Trends in Ecology & Evolution, 18, P41–P47. 10.1016/S0169-5347(02)00004-6 21236857

[bib20] Chen H., Maklakov A. (2014). Condition dependence of male mortality drives the evolution of sex differences in longevity. Current Biology, 24, 2423–2427. 10.1016/j.cub.2014.08.055 25308078

[bib21] Chippindale A., Leroi A., Kim S., Rose M. (1993). Phenotypic plasticity and selection in *Drosophila* life-history evolution. I. Nutrition and the cost of reproduction. Journal of Evolutionary Biology, 6, 171–193. 10.1046/j.1420-9101.1993.6020171.x

[bib22] Chippindale A., Leroi A., Saing H., Borash D., Rose M. (1997). Phenotypic plasticity and selection in *Drosophila* life-history evolution. II. Diet, mates and the cost of reproduction. Journal of Evolutionary Biology, 10, 269–293.

[bib23] Cho L., Yu C., Kao C. (2021). Social perception of young adults prolongs the lifespan of aged *Drosophila*. NPJ Aging and Mechanisms of Disease, 7, 21. 10.1038/s41514-021-00073-8 34471134 PMC8410773

[bib24] Clutton-Brock T., Isvaran K. (2007). Sex differences in ageing in natural populations of vertebrates. Proceedings of the Royal Society B: Biological Sciences, 274, 3097–3104. 10.1098/rspb.2007.1138 PMC2293943 17939988

[bib25] Colchero F., Jones O., Rebke M. (2012). BaSTA: An R package for Bayesian estimation of age-specific survival from incomplete mark-recapture/recovery data with covariates. Methods in Ecology and Evolution, 3, 466–470. 10.1111/j.2041-210X.2012.00186.x

[bib26] Colchero F., Rau R., Jones O. R., Barthold J. A., Conde D. A., Lenart A., Nemeth L., Scheuerlein A., Schoeley J., Torres C., Zarulli V., Altmann J., Brockman D. K., Bronikowski A. M., Fedigan L. M., Pusey A. E., Stoinski T. S., Strier K. B., Baudisch A., … Vaupel J. W. (2016). The emergence of longevous populations. Proceedings of the National Academy of Sciences, 113, E7681–E7690. 10.1073/pnas.1612191113 PMC5137748 27872299

[bib27] Cook P., Wedell N. (1996). Ejaculate dynamics in butterflies: A strategy for maximizing fertilization success?. Proceedings of the Royal Society of London. Series B: Biological Sciences, 263, 1047–1051.

[bib28] Dore A., Rostant W., Bretman A., Chapman T. (2021). Plastic male mating behavior evolves in response to the competitive environment. Evolution, 75, 101–115. 10.1111/evo.14089 32844404

[bib29] Flintham E., Yoshida T., Smith S., Pavlou H., Goodwin S., Carazo P., Wigby S. (2018). Interactions between the sexual identity of the nervous system and the social environment mediate lifespan in *Drosophila melanogaster*. Proceedings of the Royal Society B: Biological Sciences, 285, 20181450. 10.1098/rspb.2018.1450 PMC6283938 30487307

[bib30] Fowler E., Leigh S., Rostant W., Thomas A., Bretman A., Chapman T. (2022). Memory of social experience affects female fecundity via perception of fly deposits. BMC Biology, 20, 244. 10.1186/s12915-022-01438-5 36310170 PMC9620669

[bib31] Gage M. (1991). Risk of sperm competition directly affects ejaculate size in the Mediterranean fruit fly. Animal Behaviour, 42, 1036–1037. 10.1016/S0003-3472(05)80162-9

[bib32] Godin J. (2003). Predator preference for brightly colored males in the guppy: A viability cost for a sexually selected trait. Behavioral Ecology, 14, 194–200. 10.1093/beheco/14.2.194

[bib33] Graves B. (2007). Sexual selection effects on the evolution of senescence. Evolutionary Ecology, 21, 663–668. 10.1007/s10682-006-9144-6

[bib34] Harrison L., Churchill E., Fairweather M., Smithson C., Chapman T., Bretman A. (2024). Ageing effects of social environments in “non-social” insects. Philosophical Transactions of the Royal Society B: Biological Sciences, 379, 20220463. 10.1098/rstb.2022.0463 PMC11513649 39463243

[bib35] Harrison X. (2014). Using observation-level random effects to model overdispersion in count data in ecology and evolution. PeerJ, 2, e616. 25320683 10.7717/peerj.616 PMC4194460

[bib36] Harshman L., Zera A. (2007). The cost of reproduction: The devil in the details. Trends in Ecology and Evolution, 22, 80–86. 10.1016/j.tree.2006.10.008 17056152

[bib37] Hartig F. (2020). DHARMa: Residual diagnostics for hierarchical (multi-level/mixed) regression models. R package version 0.3.3.0. CRAN. https://CRAN.R-project.org/package=DHARMa

[bib38] Hoffman J., Dudeck S., Patterson H., Austad S. (2021). Sex, mating and repeatability of *Drosophila melanogaster* longevity. Royal Society Open Science, 8, 210273. 10.1098/rsos.210273 34457337 PMC8371361

[bib39] Houle D., Rowe L. (2003). Natural selection in a bottle. The American Naturalist, 161, 50–67. 10.1086/345480 12650462

[bib40] Kawasaki N., Brassil C., Brooks R., Bonduriansky R. (2008). Environmental effects on the expression of life span and aging: An extreme contrast between wild and captive cohorts of *Telostylinus angusticollis* (Diptera: Neriidae). The American Naturalist, 172, 346–357. 10.1086/589519 18710341

[bib41] Kimber C., Chippindale A. (2013). Mutation, condition, and the maintenance of extended lifespan in *Drosophila*. Current Biology, 23, P2283–P2287. 10.1016/j.cub.2013.09.049 24210612

[bib42] Lailvaux S., Irschick D. (2006). A functional perspective on sexual selection: Insights and future prospects. Animal Behaviour, 72, 263–273. 10.1016/j.anbehav.2006.02.003

[bib43] Leech T., Evison S., Armitage S., Sait S., Bretman A. (2019). Interactive effects of social environment, age and sex on immune responses in *Drosophila melanogaster*. Journal of Evolutionary Biology, 32, 1082–1092. 10.1111/jeb.13509 31313398

[bib44] Lemaître J., Berger V., Bonenfant C., Douhard M., Gamelon M., Plard F., Gaillard J. (2015). Early-late life trade-offs and the evolution of ageing in the wild. Proceedings of the Royal Society, 282, 20150209. 10.1098/rspb.2015.0209 PMC4426628 25833848

[bib45] Lemaître J., Gaillard J., Ramm S. (2020a). The hidden ageing costs of sperm competition. Ecology Letters, 23, 1573–1588. 32906225 10.1111/ele.13593

[bib46] Lemaître J., Moorad J., Gaillard J., Maklakov A., Nussey D. (2024). A unified framework for evolutionary genetic and physiological theories of aging. PLoS Biology, 22, e3002513 38412150 10.1371/journal.pbio.3002513 PMC10898761

[bib47] Lemaître J., Ronget V., Tidière M., Allainé D., Berger V., Cohas A., Colchero F., Conde D., Garratt M., Liker A., Marais G., Scheuerlein A., Székely T., Gaillard J. (2020b). Sex differences in adult lifespan and aging rates of mortality across wild mammals. Proceedings of the National Academy of Sciences of the United States of America, 117, 8546–8553. 32205429 10.1073/pnas.1911999117 PMC7165438

[bib48] Lind M., Carlsson H., Duxbury E., Ivimey-Cook E., Maklakov A. (2021). Cost-free lifespan extension via optimization of gene expression in adulthood aligns with the developmental theory of ageing. Proceedings of the Royal Society B: Biological Sciences, 288, 20201728. 10.1098/rspb.2020.1728 PMC7893226 33529563

[bib49] Lizé A., Price T., Heys C., Lewis Z., Hurst G. (2014). Extreme cost of rivalry in a monandrous species: Male–male interactions result in failure to acquire mates and reduced longevity. Proceedings of the Royal Society B: Biological Sciences, 281, 20140631 10.1098/rspb.2014.0631 PMC4046415 24827446

[bib50] Magwere T., Chapman T., Partridge L. (2004). Sex differences in the effect of dietary restriction on life span and mortality rates in female and male *Drosophila melanogaster*. Journals of Gerontology A, 59, B3–B9. 10.1093/gerona/59.1.b3 14718480

[bib51] Maklakov A., Chapman T. (2019). Evolution of ageing as a tangle of trade-offs: Energy versus function. Proceedings of the Royal Society B: Biological Sciences, 286, 20191604. 10.1098/rspb.2019.1604 PMC6784717 31530150

[bib52] Maklakov A., Fricke C., Arnqvist G. (2007). Sexual selection affects lifespan and aging in the seed beetle. Aging Cell, 6, 739–744. 10.1111/j.1474-9726.2007.00333.x 17725688

[bib53] Maklakov A., Lummaa V. (2013). Evolution of sex differences in lifespan and aging: Causes and constraints. BioEssays, 35, 717–724. 10.1002/bies.201300021 23733656

[bib54] Maures T., Booth L., Benayoun B., Izrayelit Y., Schroeder F., Brunet A. (2014). Males shorten the life span of *C. elegans* hermaphrodites via secreted compounds. Science, 343, 541–544. 10.1126/science.1244160 24292626 PMC4126796

[bib55] Moorad J., Promislow D., Silvertown J. (2019). Evolutionary ecology of senescence and a reassessment of Williams’ “extrinsic mortality” hypothesis. Trends in Ecology & Evolution, 34, 519–530. 10.1016/j.tree.2019.02.006 30857756 PMC6746179

[bib56] Partridge L., Fowler K., Trevitt S., Sharp W. (1986). An examination of the effects of males on the survival and egg-production rates of female *Drosophila melanogaster*. Journal of Insect Physiology, 32, 925–929. 10.1016/0022-1910(86)90140-X

[bib57] Pilakouta N., Halford C., Rácz R., Smiseth P. (2016). Effects of prior contest experience and contest outcome on female reproductive decisions and offspring fitness. The American Naturalist, 188, 319–328. 10.1086/687392 27501089

[bib58] Piper M., Partridge L. (2018). *Drosophila* as a model for ageing. Biochimica Et Biophysica Acta (BBA)—Molecular Basis of Disease, 1864, 2707–2717. 10.1016/j.bbadis.2017.09.016 28964875

[bib59] Promislow D. (2003). Mate choice, sexual conflict, and evolution of senescence. Behavior Genetics, 33, 191–201. 10.1023/A:1022562103669 14574152

[bib60] R Development Core Team . (2020). R: A language and environment for statistical computing.

[bib61] Reznick D. (1992). Measuring the costs of reproduction. Trends in Ecology and Evolution, 7, 42–45. 10.1016/0169-5347(92)90150-A 21235948

[bib62] Rice W. (1996). Sexually antagonistic male adaptation triggered by experimental arrest of female evolution. Nature, 381, 232–234. 10.1038/381232a0 8622764

[bib63] Rice W. (1998). Male fitness increases when females are eliminated from gene pool: Implications for the Y chromosome. Proceedings of the National Academy of Sciences, 95, 6217–6221. 10.1073/pnas.95.11.6217 PMC27633 9600945

[bib64] Rose M., Charlesworth B. (1981). Genetics of life history in *Drosophila melanogaster*. Genetics, 97, 187–196. 10.1093/genetics/97.1.187 6790341 PMC1214383

[bib65] Rostant W., Mason J., de Coriolis J., Chapman T. (2020). Resource-dependent evolution of female resistance responses to sexual conflict. Evolution Letters, 4, 54–64. 10.1002/evl3.153 32055411 PMC7006461

[bib66] Rostant W., Mason J., West N., Maklakov A., Chapman T. (2023). Socio-sexual exposure has opposing effects on male and female actuarial senescence in the fruit fly *Drosophila melanogaster*. The Journals of Gerontology: Series A, 12, 2230–2239. 10.1093/gerona/glad215 PMC10692434 37694551

[bib67] Schärer L., Rowe L., Arnqvist G. (2012). Anisogamy, chance and the evolution of sex roles. Trends in Ecology and Evolution, 27, 260–264. 10.1016/j.tree.2011.12.006 22277154

[bib68] Sgrò C., Partridge L. (2000). Evolutionary responses of the life history of wild-caught *Drosophila melanogaster* to two standard methods of laboratory culture. The American Naturalist, 156, 341–353.

[bib69] Sgrò C., Partridge L. (2001). Laboratory adaptation of life history in *Drosophila*. The American Naturalist, 158, 657–658. 10.1086/323592 18707359

[bib70] Simmons L., Craig M., Llorens T., Schinzig M., Hosken D. (1993). Bushcricket spermatophores vary in accord with sperm competition and parental investment theory. Proceedings of the Royal Society of London. Series B: Biological Sciences, 251, 183–186.

[bib71] Stearns S. (1989). Trade-offs in life-history evolution. Functional Ecology, 3, 259–268. 10.2307/2389364

[bib72] Travers L., Garcia-Gonzalez F., Simmons L. (2015). Live fast die young life history in females: Evolutionary trade-off between early life mating and lifespan in female *Drosophila melangaster*. Scientific Reports, 5, 15469. 10.1038/srep15469 26482533 PMC4612512

[bib73] Trivers R. (1972). Parental investment and sexual selection. In Campbell B. (Ed.), Sexual selection and the descent of man(pp. ​​​​​​136–179.). Aldine.

[bib74] Tuttle M., Ryan M. (1981). Bat predation and the evolution of frog vocalizations in the Neotropics. Science, 214, 677–678. 10.1126/science.214.4521.677 17839660

[bib75] Wedell N., Gage M., Parker G. (2002). Sperm competition, male prudence and sperm-limited females. Trends in Ecology & Evolution, 17, 313–320. 10.1016/S0169-5347(02)02533-8

[bib76] Williams G. (1957). Pleiotropy, natural selection, and the evolution of senescence. Evolution, 11, 398–411. 10.1111/j.1558-5646.1957.tb02911.x

[bib77] Yun L., Chen P., Singh A., Agrawal A., Rundle H. (2017). The physical environment mediates male harm and its effect on selection in females. Proceedings of the Royal Society B: Biological Sciences, 284, 20170424. 10.1098/rspb.2017.0424 PMC5524491 28679725

[bib78] Zajitschek F., Bonduriansky R., Zajitschek S., Brooks R. (2009). Sexual dimorphism in life history: Age, survival, and reproduction in male and female field crickets *Teleogryllus commodus* under seminatural conditions. The American Naturalist, 173, 792–802. 10.1086/598486 19374505

