# Supplementary material for: Fast females, slow males: accelerated ageing and reproductive senescence in Drosophila melanogaster females across diverse social environments
Source: Evol Lett. 2025 Nov 8;10(1):42–53. doi: 10.1093/evlett/qraf041 (PMC12870849; doi:10.1093/evlett/qraf041)
Supplement: qraf041_Supplemental_Files [file qraf041_supplemental_files.zip › Supp_material.docx]

**Supporting Information**

**This file includes:**

Figures S1 to S3

Tables S1 to S9

**Other supporting materials for this manuscript include the following:**

Datasets S1 to S9

Code


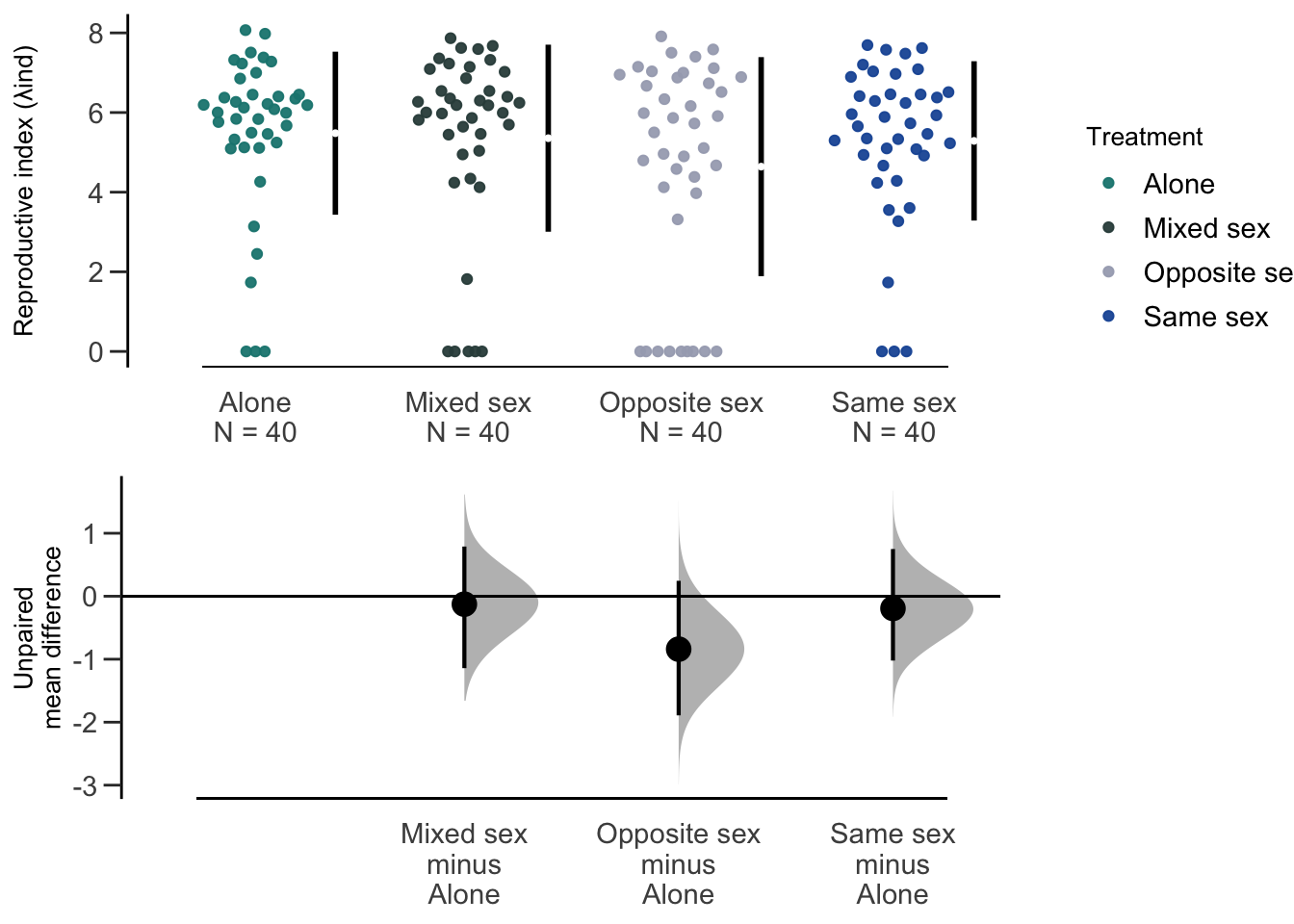


**Fig. S1.** Male individual fitness (lambda_ind_) for the four social treatments. The first set of plots depict the raw data, their mean and standard deviation across the four social treatments. The second set of plots compare the mean effect sizes for each of the four social treatments (mean and 95% confidence intervals are shown). Plots were generated using the R package *dabestR.*


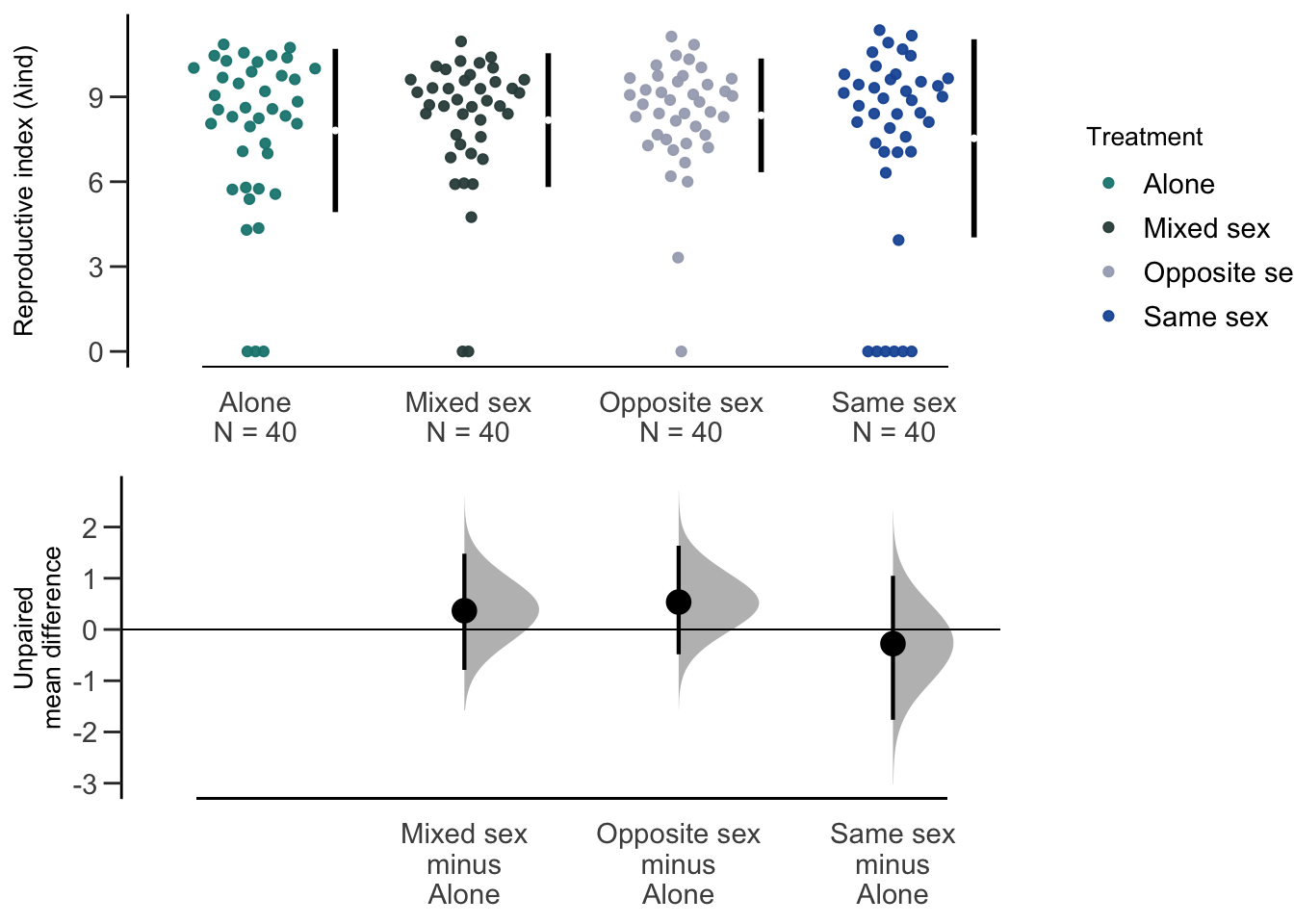


**Fig. S2.** Female individual fitness (lambda_ind_) for the four social treatments. The first set of plots depict the raw data, their mean and standard deviation across the four social treatments. The second set of plots compare the mean effect sizes for each of the four social treatments (mean and 95% confidence intervals are shown). Plots were generated using the R package *dabestR.*


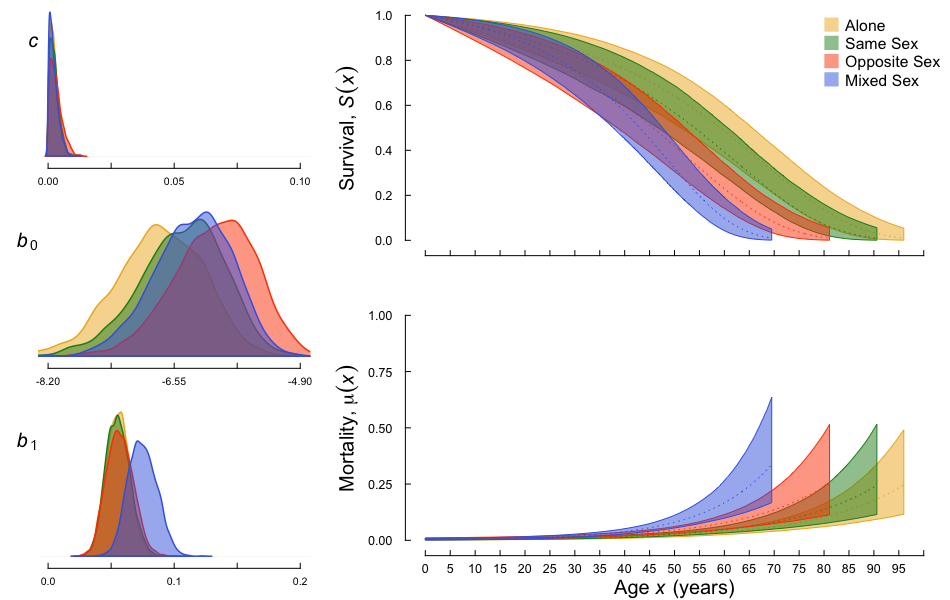

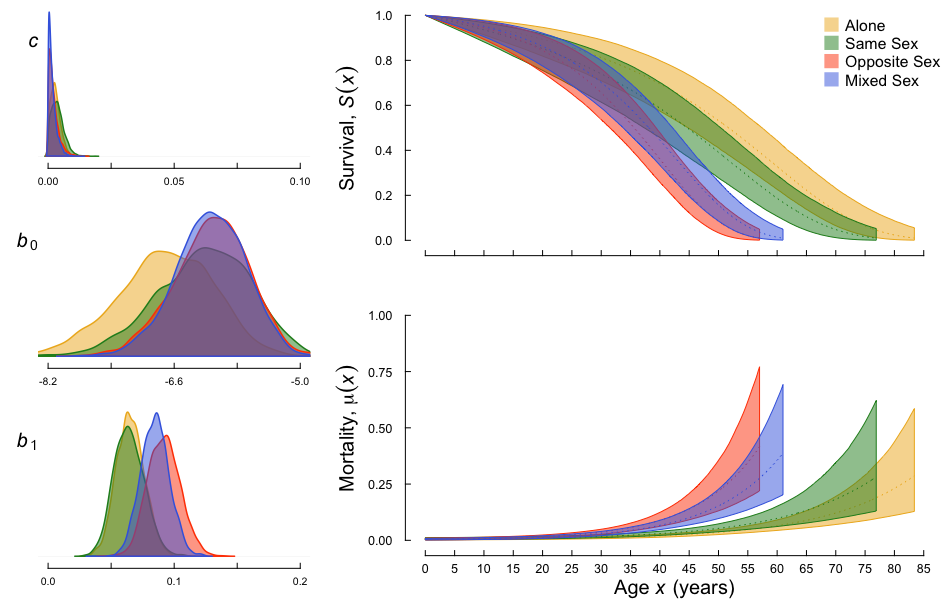


**B**

**A**

Age (days)

Age (days)

**Fig. S3.** Plots depicting ageing and mortality parameters and patterns generated from *BaSTA* models for males **(A)** and females **(B)** in the four different social environment treatments. Plot *c* shows the Gompertz mortality rate parameter, while plots *b*_0_ and *b*_1_ show the baseline and age-specific mortality parameters, respectively. Survival curves and mortality rate plots depict overall lifespan and ageing patterns.

**Table S1. Summary statistics for male and female lifespan in each of the four social treatments.**

| Treatment | Mean Survival (days) | Standard Deviation | Sample Size |
| --- | --- | --- | --- |
| *Males* |  |  |  |
| Alone | 59.6 | 23.1 | 40 |
| Mixed sex | 43.8 | 14.6 | 40 |
| Opposite Sex | 46.5 | 19.7 | 40 |
| Same sex | 54.5 | 21.8 | 40 |
| *Females* |  |  |  |
| Alone | 51.6 | 20.8 | 40 |
| Mixed sex | 38.9 | 10.9 | 40 |
| Opposite Sex | 36.1 | 11.1 | 40 |
| Same sex | 44.6 | 19.7 | 40 |

Table S2. Cox proportional hazards model output for males and females. Each model compares the likelihood of survival to the control social treatment: Alone. Treatments that significantly increase the likelihood of mortality are highlighted in bold (positive values indicate higher likelihood of death).

| Treatment | Coefficient | Standard Error | *z* | *P*-value |
| --- | --- | --- | --- | --- |
| *Males* |  |  |  |  |
| Mixed sex | 1.84 | 0.29 | 6.37 | <0.0001 |
| Opposite sex | 1.07 | 0.27 | 3.92 | <0.0001 |
| Same sex | 0.47 | 0.25 | 1.88 | 0.06 |
| *Females* |  |  |  |  |
| Mixed sex | 1.69 | 0.27 | 6.23 | <0.0001 |
| Opposite sex | 1.95 | 0.28 | 4.07 | <0.0001 |
| Same sex | 0.66 | 0.24 | 1.21 | 0.006 |

Table S3. Pairwise comparisons (i.e., mean Kullback-Leibler discrepancy calibrations (KLDC)) for each treatment and for each of the three mortality parameters obtained from *BaSTA* models. Significant differences (values greater than 0.8) between treatments are highlighted in bold.

| Mean Kullback-Leibler discrepancy calibration (KLDC) | c  (Makeham mortality parameter) | b0  (baseline mortality) | b1  (Gompertz mortality) |
| --- | --- | --- | --- |
| *Males* |  |  |  |
| Same sex – Alone | 0.51 | 0.56 | 0.50 |
| Opposite sex – Alone | 0.62 | 0.79 | 0.51 |
| Opposite sex – Same sex | 0.56 | 0.63 | 0.51 |
| **Mixed sex – Alone** | 0.50 | 0.65 | **0.92** |
| **Mixed sex – Same sex** | 0.50 | 0.52 | **0.91** |
| **Mixed sex – Opposite sex** | 0.59 | 0.57 | **0.87** |
| *Females* |  |  |  |
| Same sex – Alone | 0.57 | 0.64 | 0.51 |
| **Opposite sex – Alone** | 0.53 | 0.74 | **0.96** |
| **Opposite sex – Same sex** | 0.64 | 0.54 | **0.96** |
| **Mixed sex – Alone** | 0.63 | 0.76 | **0.89** |
| **Mixed sex – Same sex** | 0.77 | 0.55 | **0.89** |
| Mixed sex – Opposite sex | 0.54 | 0.50 | 0.59 |

Table S4. Full model output for males and females when testing for effects of the social environment on individual fitness (lambda_ind_).

| Model Terms | Estimate | *SE* | *z* | *P-*value |
| --- | --- | --- | --- | --- |
| *Males* |  |  |  |  |
| **Random effects** | **Variance** | ***SD*** |  |  |
| Block | 0.01 | 0.08 |  |  |
| **Conditional model** |  |  |  |  |
| **Intercept (Alone)** | 1.77 | 0.05 | 33.24 | **<0.0001** |
| Mixed sex | 0.03 | 0.05 | 0.56 | 0.573 |
| Opposite sex | 0.01 | 0.05 | 0.16 | 0.870 |
| Same sex | -0.03 | 0.05 | -0.62 | 0.537 |
| **Zero-inflated model** |  |  |  |  |
| **Intercept** | -1.95 | 0.24 | -8.14 | **<0.0001** |
| *Females* |  |  |  |  |
| **Random effects** | **Variance** | ***SD*** |  |  |
| Block | 0.01 | 0.09 |  |  |
| **Conditional model** |  |  |  |  |
| **Intercept (Alone)** | 2.12 | 0.05 | 41.43 | **<0.0001** |
| Mixed sex | 0.02 | 0.04 | 0.53 | 0.595 |
| Opposite sex | 0.02 | 0.04 | 0.56 | 0.578 |
| Same sex | 0.05 | 0.04 | 1.35 | 0.177 |
| **Zero-inflated model** |  |  |  |  |
| **Intercept** | -2.51 | 0.30 | -8.37 | **<0.0001** |

Table S5. Model summaries for age-specific reproduction for both males and females. Models include a quadratic term for age. Significant effects are highlighted in bold.

| Model Terms | Estimate | *SE* | *z* | *P-*value |
| --- | --- | --- | --- | --- |
| *Males* |  |  |  |  |
| **Random effects** | **Variance** | ***SD*** |  |  |
| Block | 0.021 | 0.014 |  |  |
| Fly ID | <0.0001 | <0.0001 |  |  |
| **Conditional model** |  |  |  |  |
| **Intercept (Alone)** | 3.49 | 0.13 | 27.44 | **<0.0001** |
| Mixed sex | -0.004 | 0.18 | -0.02 | 0.983 |
| Opposite sex | -0.04 | 0.18 | -0.25 | 0.805 |
| Same sex | -0.07 | 0.15 | -0.47 | 0.642 |
| Age | -0.01 | 0.05 | -0.21 | 0.838 |
| Age^2^ | -0.00003 | 0.005 | -0.005 | 0.996 |
| Mixed sex*Age | 0.05 | 0.11 | 0.45 | 0.654 |
| Opposite sex*Age | 0.03 | 0.10 | 0.322 | 0.747 |
| Same sex* Age | 0.008 | 0.07 | 0.10 | 0.920 |
| Mixed sex*Age^2^ | -0.007 | 0.01 | -0.49 | 0.624 |
| Opposite sex*Age^2^ | -0.003 | 0.01 | -0.23 | 0.817 |
| Same sex*Age^2^ | 0.0007 | 0.008 | 0.09 | 0.932 |
| **Zero-inflated model** |  |  |  |  |
| **Intercept** | -2.05 | 0.46 | -4.49 | **<0.0001** |
| Mixed sex | -0.84 | 0.56 | -1.49 | 0.136 |
| Opposite sex | 0.25 | 0.51 | 0.49 | 0.623 |
| Same sex | -0.20 | 0.49 | -0.41 | 0.686 |
| Age | -0.02 | 0.16 | -0.13 | 0.893 |
| **Age^2^** | 0.03 | 0.01 | 2.19 | **0.029** |
| **Mixed sex*Age** | 0.43 | 0.12 | 3.74 | **0.0002** |
| **Opposite sex*Age** | 0.49 | 0.12 | 4.08 | **<0.0001** |
| Same sex*Age | 0.01 | 0.08 | 0.07 | 0.943 |
| *Females* |  |  |  |  |
| **Random effects** | **Variance** | ***SD*** |  |  |
| Block | 0.01 | 0.11 |  |  |
| Fly ID | 0.04 | 0.20 |  |  |
| **Conditional model** |  |  |  |  |
| **Intercept (Alone)** | 4.05 | 0.12 | 33.75 | **<0.0001** |
| Mixed sex | 0.20 | 0.16 | 1.27 | 0.204 |
| Opposite sex | 0.14 | 0.18 | 0.80 | 0.424 |
| **Same sex** | 0.34 | 0.15 | 2.22 | **0.026** |
| **Age** | 0.18 | 0.07 | 2.65 | **0.008** |
| **Age^2^** | -0.06 | 0.01 | -6.76 | **<0.0001** |
| Mixed sex*Age | -0.17 | 0.11 | -1.56 | 0.119 |
| Opposite sex*Age | -0.07 | 0.14 | -0.48 | 0.633 |
| **Same sex*Age** | -0.24 | 0.10 | -2.53 | **0.011** |
| Mixed sex*Age^2^ | 0.02 | 0.02 | 1.07 | 0.283 |
| Opposite sex*Age^2^ | -0.02 | 0.03 | -0.73 | 0.466 |
| **Same sex*Age^2^** | 0.04 | 0.01 | 2.84 | **0.005** |
| **Zero-inflated model** |  |  |  |  |
| **Intercept** | -3.14 | 0.75 | -4.17 | **<0.0001** |
| Mixed sex | 0.05 | 0.80 | 0.07 | 0.946 |
| Opposite sex | -2.37 | 1.09 | -2.18 | 0.029 |
| Same sex | 0.62 | 0.73 | 0.85 | 0.397 |
| Age | -0.09 | 0.27 | -0.34 | 0.735 |
| **Age^2^** | 0.08 | 0.03 | 2.91 | **0.004** |
| Mixed sex*Age | 0.17 | 0.16 | 1.06 | 0.289 |
| **Opposite sex*Age** | 0.90 | 0.24 | 3.76 | **0.0002** |
| Same sex*Age | -0.11 | 0.13 | -0.84 | 0.400 |

Table S6. Model summaries for observed courtship effort for males and females during the weekly mating assays. Significant effects are highlighted in bold.

| Model Terms | Estimate | *SE* | *z* | *P-*value |
| --- | --- | --- | --- | --- |
| *Males* |  |  |  |  |
| **Random effects** | **Variance** | ***SD*** |  |  |
| Block | <0.001 | <0.001 |  |  |
| Fly ID | <0.001 | <0.001 |  |  |
| Observations | <0.001 | <0.001 |  |  |
| **Conditional model** |  |  |  |  |
| Intercept (Alone) | 0.71 | 0.38 | 1.87 | 0.061 |
| Mixed sex | -0.69 | 0.62 | -1.11 | 0.267 |
| Opposite sex | -0.37 | 0.59 | -0.63 | 0.531 |
| **Same sex** | 1.36 | 0.55 | 2.48 | **0.013** |
| **Age** | 0.32 | 0.14 | 2.31 | **0.021** |
| **Age^2^** | -0.03 | 0.01 | -2.46 | **0.014** |
| Mixed sex*Age | 0.26 | 0.28 | 0.94 | 0.346 |
| Opposite sex*Age | 0.05 | 0.24 | 0.22 | 0.826 |
| **Same sex*Age** | -0.41 | 0.21 | -1.97 | **0.049** |
| Mixed sex*Age^2^ | -0.03 | 0.03 | -0.97 | 0.334 |
| Opposite sex*Age^2^ | 0.00 | 0.02 | 0.01 | 0.992 |
| Same sex*Age^2^ | 0.03 | 0.02 | 1.70 | 0.089 |
| **Zero-inflated model** |  |  |  |  |
| **Intercept** | -1.25 | 0.40 | -3.12 | **0.002** |
| Mixed sex | 0.31 | 0.35 | 0.90 | 0.367 |
| **Opposite sex** | 0.75 | 0.30 | 2.52 | **0.012** |
| Same sex | -0.03 | 0.30 | -0.11 | 0.911 |
| **Age** | -0.39 | 0.14 | -2.73 | **0.006** |
| **Age^2^** | 0.04 | 0.01 | 3.28 | **0.001** |
| *Females* |  |  |  |  |
| **Random effects** | **Variance** | ***SD*** |  |  |
| Block | 0.03 | 0.17 |  |  |
| Fly ID | 0.02 | 0.14 |  |  |
| Observations | <0.0001 | <0.0001 |  |  |
| **Conditional model** |  |  |  |  |
| **Intercept (Alone)** | 1.59 | 0.26 | 6.14 | **<0.0001** |
| Mixed sex | 0.44 | 0.36 | 1.22 | 0.223 |
| Opposite sex | 0.31 | 0.36 | 0.85 | 0.393 |
| Same sex | -0.22 | 0.36 | -0.62 | 0.533 |
| Age | 0.13 | 0.10 | 1.39 | 0.165 |
| Age^2^ | -0.002 | 0.01 | -0.28 | 0.782 |
| Mixed sex*Age | -0.23 | 0.17 | -1.34 | 0.182 |
| Opposite sex*Age | -0.07 | 0.17 | -0.34 | 0.698 |
| Same sex* Age | 0.08 | 0.15 | 0.55 | 0.585 |
| Mixed sex*Age^2^ | 0.03 | 0.02 | 1.37 | 0.172 |
| Opposite sex*Age^2^ | 0.01 | 0.02 | 0.57 | 0.566 |
| Same sex*Age^2^ | -0.01 | 0.01 | -0.50 | 0.616 |
| **Zero-inflated model** |  |  |  |  |
| Intercept | -0.70 | 0.46 | -1.51 | 0.132 |
| Mixed sex | 0.43 | 0.38 | 1.14 | 0.253 |
| Opposite sex | 0.52 | 0.37 | 1.38 | 0.168 |
| Same sex | 0.01 | 0.39 | 0.02 | 0.984 |
| Age | -0.31 | 0.23 | -1.36 | 0.174 |
| Age^2^ | -0.001 | 0.03 | -0.04 | 0.970 |

**Table S7.** Model summaries for the number of observed matings for males and females during the weekly mating assays. Significant effects are highlighted in bold.

| Model Terms | Estimate | *SE* | *z* | *P-*value |
| --- | --- | --- | --- | --- |
| *Males* |  |  |  |  |
| **Random effects** | **Variance** | ***SD*** |  |  |
| Block | 0.10 | 0.32 |  |  |
| Fly ID | 1.05 | 1.03 |  |  |
| **Conditional model** |  |  |  |  |
| **Intercept (Alone)** | -1.85 | 0.86 | -2.14 | **0.033** |
| Mixed sex | -0.62 | 0.92 | -0.67 | 0.503 |
| Opposite sex | -1.78 | 1.14 | -1.56 | 0.118 |
| Same sex | -0.98 | 0.92 | -1.07 | 0.285 |
| Age | -0.05 | 0.12 | -0.42 | 0.675 |
| Mixed sex*Age | 0.11 | 0.19 | 0.59 | 0.559 |
| Opposite sex*Age | 0.42 | 0.22 | 1.91 | 0.056 |
| Same sex* Age | 0.11 | 0.16 | 0.71 | 0.478 |
| **Zero-inflated model** |  |  |  |  |
| **Intercept** | -2.87 | 0.32 | -8.93 | **<0.0001** |
| Mixed sex | 0.43 | 0.30 | 1.46 | 0.143 |
| **Opposite sex** | 1.62 | 0.27 | 6.09 | **<0.0001** |
| Same sex | -0.24 | 0.26 | -0.93 | 0.354 |
| **Age** | 0.30 | 0.04 | 7.74 | **<0.0001** |
| *Females* |  |  |  |  |
| **Random effects** | **Variance** | ***SD*** |  |  |
| Block | 0.09 | 0.31 |  |  |
| Fly ID | 0.04 | 0.20 |  |  |
| **Conditional model** |  |  |  |  |
| Intercept (Alone) | -0.27 | 0.35 | -0.76 | 0.449 |
| Mixed sex | -0.67 | 0.57 | -1.17 | 0.241 |
| **Opposite sex** | -3.53 | 0.99 | -3.58 | **0.0003** |
| Same sex | -0.63 | 0.47 | -1.36 | 0.175 |
| **Age** | -0.21 | 0.07 | -3.19 | **0.001** |
| Mixed sex*Age | -0.06 | 0.16 | -0.39 | 0.693 |
| **Opposite sex*Age** | 0.56 | 0.21 | 2.68 | **0.007** |
| Same sex*Age | 0.12 | 0.10 | 1.22 | 0.223 |

Table S8. Model summaries for activity rates of males and females during the weekly mating assays. Significant effects are highlighted in bold.

| Model Terms | Estimate | *SE* | *z* | *P-*value |
| --- | --- | --- | --- | --- |
| *Males* |  |  |  |  |
| **Random effects** | **Variance** | ***SD*** |  |  |
| Block | 0.004 | 0.06 |  |  |
| Fly ID | 0.03 | 0.17 |  |  |
| Observations | <0.0001 | <0.0001 |  |  |
| **Conditional model** |  |  |  |  |
| **Intercept (Alone)** | **3.61** | **0.12** | **29.60** | **<0.0001** |
| Mixed sex | -0.13 | 0.20 | -0.65 | 0.517 |
| Opposite sex | 0.08 | 0.19 | 0.44 | 0.662 |
| **Same sex** | **-0.48** | **0.17** | **-2.81** | **0.005** |
| Age | -0.01 | 0.02 | -0.86 | 0.387 |
| Mixed sex*Age | -0.05 | 0.04 | -1.31 | 0.191 |
| **Opposite sex*Age** | **-0.07** | **0.03** | **-2.19** | **0.029** |
| Same sex*Age | 0.05 | 0.03 | 1.91 | 0.056 |
| *Females* |  |  |  |  |
| **Random effects** | **Variance** | ***SD*** |  |  |
| Block | 0.01 | 0.08 |  |  |
| Fly ID | 0.05 | 0.22 |  |  |
| Observations | <0.0001 | <0.0001 |  |  |
| **Conditional model** |  |  |  |  |
| **Intercept (Alone)** | 3.40 | 0.21 | 16.17 | **<0.0001** |
| Mixed sex | 0.36 | 0.30 | 1.23 | 0.221 |
| Opposite sex | -0.18 | 0.31 | -0.59 | 0.556 |
| Same sex | 0.27 | 0.29 | 0.92 | 0.356 |
| Age | 0.16 | 0.09 | 1.82 | 0.069 |
| **Age^2^** | -0.02 | 0.01 | -2.93 | **0.003** |
| Mixed sex*Age | -0.17 | 0.14 | -1.20 | 0.232 |
| Opposite sex*Age | 0.09 | 0.16 | 0.57 | 0.571 |
| Same sex*Age | -0.21 | 0.13 | -1.59 | 0.112 |
| Mixed sex*Age^2^ | 0.01 | 0.02 | 0.91 | 0.362 |
| Opposite sex*Age^2^ | -0.02 | 0.02 | -1.27 | 0.203 |
| Same sex*Age^2^ | 0.02 | 0.01 | 1.55 | 0.120 |
| **Zero-inflated model** |  |  |  |  |
| **Intercept** | -4.66 | 0.69 | -6.73 | **<0.0001** |
| **Age** | 0.24 | 0.12 | 2.05 | **0.040** |

Table S9. Model summaries for age-specific reproduction of males and females including additional progeny counts from Weeks 1, 3, 6, 7 and 10. Significant effects are highlighted in bold.

| Model Terms | Estimate | *SE* | *z* | *P-*value |
| --- | --- | --- | --- | --- |
| *Males* |  |  |  |  |
| **Random effects** | **Variance** | ***SD*** |  |  |
| Block | <0.0001 | <0.0001 |  |  |
| Fly ID | <0.0001 | <0.0001 |  |  |
| **Conditional model** |  |  |  |  |
| **Intercept (Alone / Early)** | 4.48 | 1.00 | 45.08 | **<0.0001** |
| **Mixed sex** | 1.03 | 0.13 | 8.19 | **<0.0001** |
| **Opposite sex** | 1.27 | 0.13 | 10.05 | **<0.0001** |
| Same sex | -0.02 | 0.13 | -0.19 | 0.845 |
| Mid-Life Reproduction | -0.07 | 0.14 | -0.52 | 0.604 |
| Late-Life Reproduction | -0.22 | 0.15 | -1.50 | 0.132 |
| **Mixed sex*Mid** | -1.23 | 0.20 | -6.26 | **<0.0001** |
| **Opposite sex*Mid** | -1.28 | 0.22 | -5.74 | **<0.0001** |
| Same sex*Mid | -0.04 | 0.19 | -0.23 | 0.821 |
| **Mixed sex*Late** | -2.11 | 0.27 | -7.90 | **<0.0001** |
| **Opposite sex*Late** | -1.21 | 0.27 | -4.42 | **<0.0001** |
| Same sex*Late | -0.03 | 0.21 | -0.13 | 0.896 |
| **Zero-inflated model** |  |  |  |  |
| **Intercept (Alone)** | -1.10 | 0.21 | -5.21 | **<0.0001** |
| **Mixed sex** | 0.73 | 0.28 | 2.59 | **0.010** |
| **Opposite sex** | 1.17 | 0.28 | 4.18 | **<0.0001** |
| Same sex | -0.00002 | 0.30 | 0.00 | 0.686 |
| *Females* |  |  |  |  |
| **Random effects** | **Variance** | ***SD*** |  |  |
| Block | 0.001 | 0.03 |  |  |
| Fly ID | <0.0001 | 0.0004 |  |  |
| **Conditional model** |  |  |  |  |
| **Intercept (Alone / Early)** | 5.75 | 0.11 | 51.97 | **<0.0001** |
| Mixed sex | -0.07 | 0.15 | -0.48 | 0.634 |
| Opposite sex | -0.16 | 0.15 | -1.03 | 0.301 |
| Same sex | -0.11 | 0.16 | -0.71 | 0.479 |
| **Mid-Life Reproduction** | -0.94 | 0.17 | -5.67 | **<0.0001** |
| **Late-Life Reproduction** | -2.91 | 0.18 | -15.94 | **<0.0001** |
| Mixed sex*Mid | -0.41 | 0.24 | -1.74 | 0.081 |
| **Opposite sex*Mid** | -1.00 | 0.24 | -4.14 | **<0.0001** |
| Same sex*Mid | -0.04 | 0.23 | -0.18 | 0.857 |
| Mixed sex*Late | -0.02 | 0.43 | -0.05 | 0.957 |
| Opposite sex*Late | -16.89 | 306.11 | -0.06 | 0.957 |
| **Same sex*Late** | 0.55 | 0.27 | 2.07 | **0.038** |
| **Zero-inflated model** |  |  |  |  |
| **Intercept (Early)** | -3.09 | 0.39 | -4.17 | **<0.0001** |
| **Mid** | 2.18 | 0.42 | 0.07 | **<0.0001** |
| **Late** | 3.51 | 0.43 | -2.18 | **<0.0001** |

**Datasets S1-S9 (separate file).** Datasets associated with ‘Fast females, slow males: accelerated ageing and reproductive senescence in *Drosophila melanogaster* females across diverse social environments’**.** Each dataset is on a new sheet with the first sheet containing metadata.

**Code (separate file).** R code (annotated) used for data analysis.
